# Structure-based design and discovery of novel anti-tissue factor antibodies with cooperative double-point mutations, using interaction analysis

**DOI:** 10.1038/s41598-020-74545-4

**Published:** 2020-10-16

**Authors:** Shuntaro Chiba, Aki Tanabe, Makoto Nakakido, Yasushi Okuno, Kouhei Tsumoto, Masateru Ohta

**Affiliations:** 1 Medical Sciences Innovation Hub Program, RIKEN, 1-7-22, Suehiro-cho, Tsurumi-ku, Yokohama, 230-0045 Japan; 2 Department of Bioengineering, School of Engineering, The University of Tokyo, Bunkyo-ku, Tokyo, 113-8656 Japan; 3 Graduate School of Medicine, Kyoto University, Sakyo-ku, Kyoto, 606-8507 Japan; 4 The Institute of Medical Science, The University of Tokyo, Minato-ku, Tokyo, 108-8639 Japan

**Keywords:** Molecular modelling, Protein design, Protein structure predictions

## Abstract

The generation of a wide range of candidate antibodies is important for the successful development of drugs that simultaneously satisfy multiple requirements. To find cooperative mutations and increase the diversity of mutants, an in silico double-point mutation approach, in which 3D models of all possible double-point mutant/antigen complexes are constructed and evaluated using interaction analysis, was developed. Starting from an antibody with very high affinity, four double-point mutants were designed in silico. Two of the double-point mutants exhibited improved affinity or affinity comparable to that of the starting antibody. The successful identification of two active double-point mutants showed that a cooperative mutation could be found by utilizing information regarding the interactions. The individual single-point mutants of the two active double-point mutants showed decreased affinity or no expression. These results suggested that the two active double-point mutants cannot be obtained through the usual approach i.e. a combination of improved single-point mutants. In addition, a triple-point mutant, which combines the distantly located active double-point mutation and an active single-point mutation collaterally obtained in the process of the double-point mutation strategy, was designed. The triple-point mutant showed improved affinity. This finding suggested that the effects of distantly located mutations are independent and additive. The double-point mutation approach using the interaction analysis of 3D structures expands the design repertoire for mutants, and hopefully paves a way for the identification of cooperative multiple-point mutations.

## Introduction

Antibodies selectively bind to specific molecules, and hence can be used as therapeutic and diagnostic agents^1,2^. The initial antibodies obtained through immunization or in vitro protein evolution methods, such as phage display and yeast display commonly require further optimization. Experimental and computational methods for improving affinity have been extensively studied^3–18^.

To obtain antibodies satisfying multiple properties, such as affinity and stability, a variety of mutants needs to be prepared, because such properties depend upon the amino acid sequence, and diversity of sequences is thought to increase the probability of obtaining antibodies, simultaneously satisfying the multiple properties required for use as a drug. One of the approaches used to increase the diversity of mutants is a double-point (DP) mutation, wherein two amino acids are simultaneously mutated. Because the number of cases of DP mutations is large, in silico methods are expected to identify promising DP mutations and reduce experimental cost. Although the in silico antibody design method using single-point (SP) mutations has been investigated^10–17^, in silico approaches explicitly targeting DP mutations have not been frequently reported^11,12,15,17^. Of the DP mutant approaches reported, most showed decreased affinity, except for one, which had a 1.4 ± 0.2-fold increase in affinity^12^. A key factor for success in a DP mutation approach is to find sets of two amino acid pairs, which act cooperatively, from among a large number of possible DP mutants. Affinity appears to involve positive interactions between two mutated amino acids and their surroundings, including the antigen. Molecular interactions, such as protein–protein, protein-DNA, and protein–ligand interactions at an atomic level are generally investigated by identifying their interactions, such as hydrogen bonds and van der Waals interactions, based on their 3D structures. In addition to the widely recognized hydrogen bond and van der Waals interactions, a variety of weak interactions, such as CH–π, CH–O, S–O, and S–π interactions, are important for the structure-based drug design of small molecules^19,20^.

In this study, an in silico DP mutation approach was used with the interaction analysis to examine whether cooperative DP mutants could be found and whether mutants that are more diverse could be obtained. The anti-tissue factor antibody D3h44^21^ was selected as a target protein for designing the mutants and defined as a wild-type (WT) for the following reasons: (a) the crystallographic structure of the tissue factor (TF) and the Fab of the D3h44 complex has been solved (PDB ID: 1JPS^22^), and its resolution (1.85 Å) is sufficient for the recognition of the interactions between antigen, antibody, and water; and (b) the affinity between TF and D3h44 (100 pM) has been investigated^21^.

To increase the diversity of the DP mutants and to increase the probability of finding cooperative mutants, the amino acids that did not contact the antigen but were adjacent to amino acids close to the antigen, were included as candidate residues for a mutation, in addition to the amino acids of the antibody in contact with the antigen. After defining the candidate residues for a mutation, a 3D model of all possible DP mutants was constructed. All possible DP mutants included all possible SP mutants, because a DP mutant becomes an SP mutant if one of the mutated residues is the same as the residue of the WT.

After energy-based selection of the 3D models for a visual inspection, the differences of the interactions, which include weak interactions, such as CH–π, CH–O, S–O, and S–π^23–26^ in addition to hydrogen bond and van der Waals interactions, between the WT/antigen and the DP mutant/antigen complexes were identified. Then, the interactions introduced, lost, or preserved by the mutation were manually checked and visually evaluated. Based on this evaluation, ten mutants, including SP mutants that were collaterally obtained because of the DP mutation strategy, were selected. The mutants were expressed and experimentally evaluated for affinity and stability compared to the WT. Here, we report the details of the procedures and the results of the DP mutation strategy, together with the interaction analysis for finding cooperative mutations.

## Results

### Design of mutants

**Figure 1:**
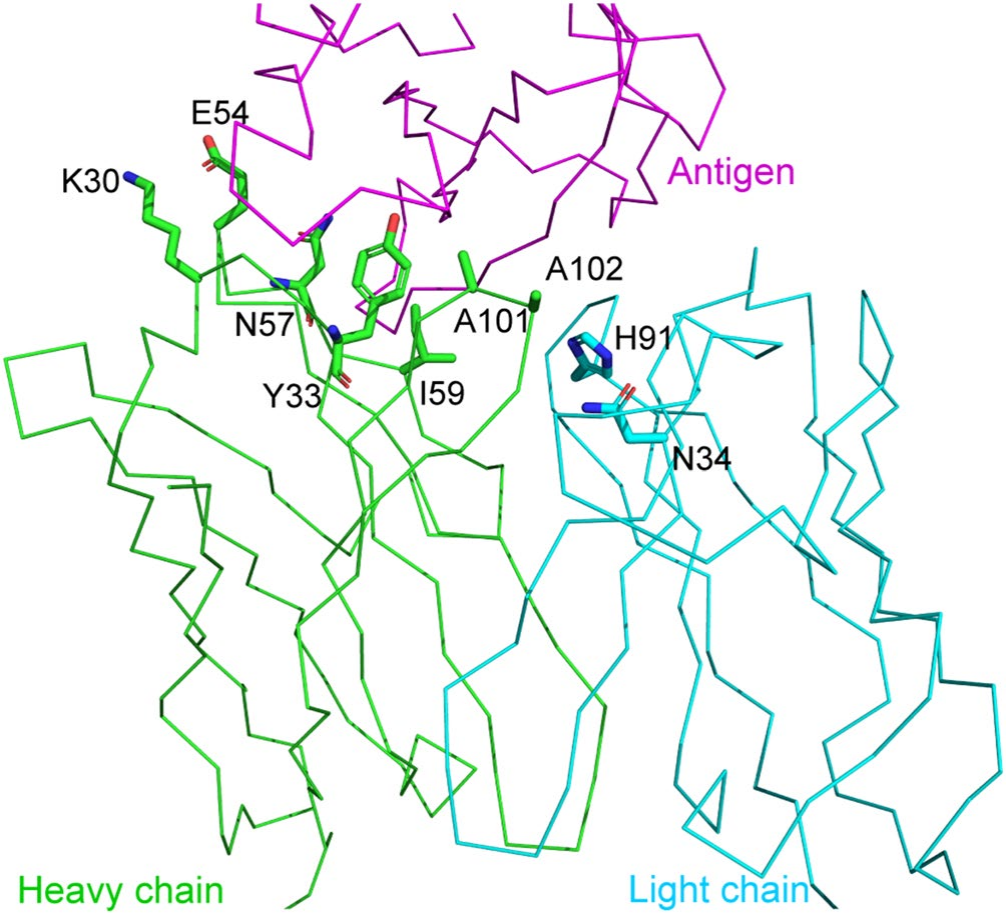
Location of target residues of four double-point and six single-point mutants. The 3D structure shown is PDB ID = 1JPS^22^. The main chain is depicted in a ribbon representation. The side chain of mutated residues is depicted in thick stick form. The heavy and the light chains of D3h44 are colored green and cyan, respectively. The antigen tissue factor is colored magenta.

Four DP mutants (L-N34D/L-H91S, H-K30Q/H-E54H, H-Y33Q/H-A101R, H-A101S/H-A102H) and six SP mutants (L-H91N, L-H91Q, L-H91E, H-N57Y, H-I59H, H-A101V) were selected for further expression and evaluation of their affinity. Although the main objective of this research was to obtain DP mutants with cooperative mutations, six SP mutants were included, because the SP mutants were generated as a by-product of the DP mutation approach. The light and heavy chains of the antibodies are denoted as “L-” and “H-” respectively. Figure 1 shows the location of the residues to be mutated. In addition to the residues of the antibody that directly contact the antigen, residues not directly contacting the antigen, but adjacent to the residues of the antibody in contact with the antigen, such as L-N34, were included (Fig. 1).

In order to assess the applicability of the modeling method for each mutation pair, the root-mean-square deviation (RMSD) of the heavy atoms of the mutation and the surrounding residues (within 4.5 Å) between those of the WT crystallographic structure and the model was calculated as described in “Methods”. The residue pairs with a model structure largely deviated from the WT structure (RMSD > 0.5 Å), which corresponded to 14% of all mutation positions, were discarded because a large structural change of the WT model will deteriorate the reliability of the mutant models for such pairs.

Before the interaction analysis, the 3D models to be visually inspected were selected based on molecular mechanics/generalized Born volume integration (MM/GBVI) energy^27^ of the 3D models of the antigen/DP mutant complex. Regarding the favorable combination of amino acids in MM/GBVI energy score, it was observed that some combinations of amino acids, such as His-Trp, Glu-Asp, and Glu-Tyr, tend to have a higher rank in the MM/GBVI score (Fig. S1). Despite this trend, the four DP mutations finally selected after the interaction analysis were not from the higher-ranked combinations in the MM/GBVI score.

**Figure 2:**
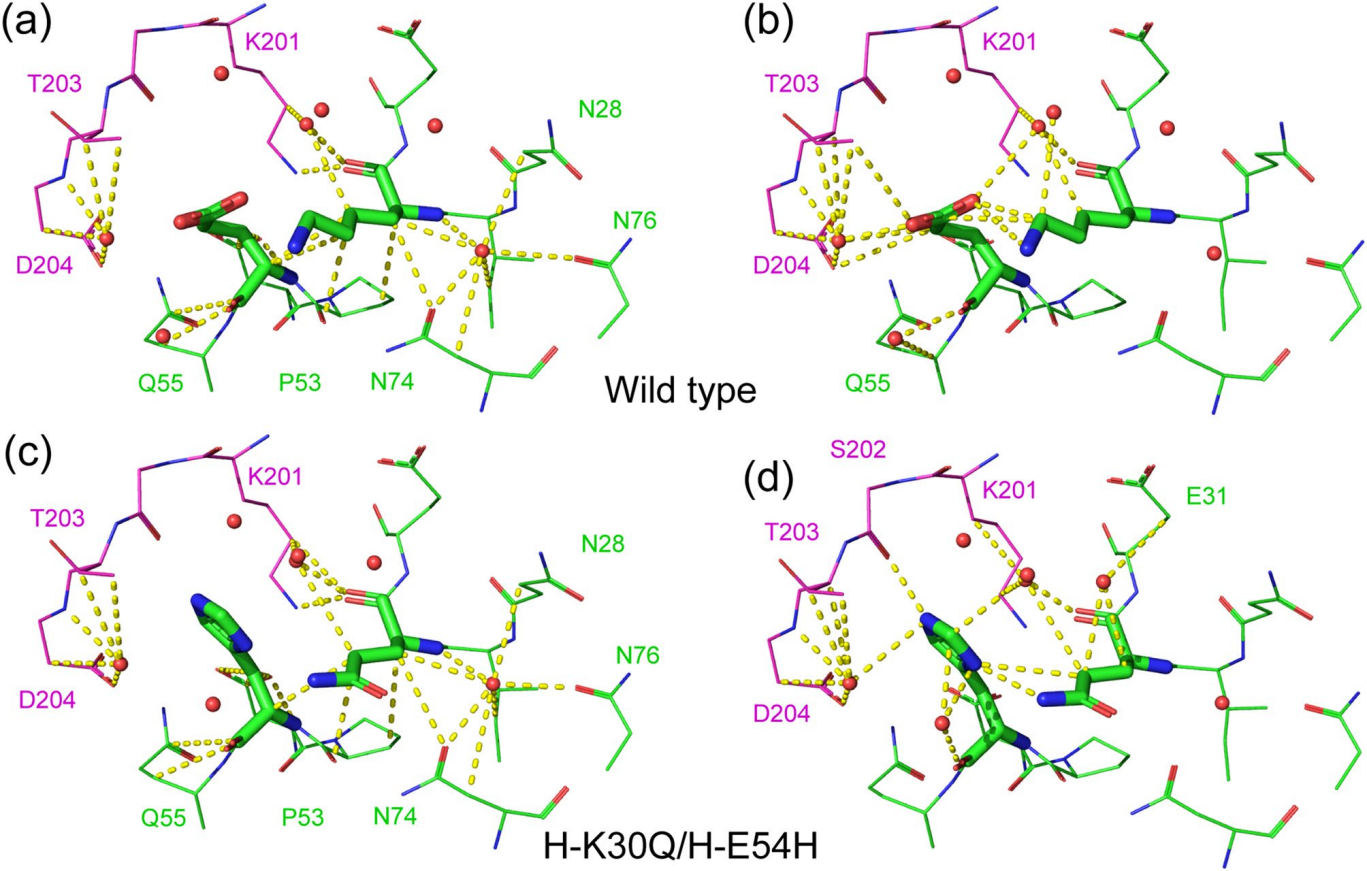
Interactions in the wild-type (WT) (PDB ID: 1JPS^22^) and the double-point mutant model. (**a**) The interactions common to the WT and the mutant are depicted in the WT structure. (**b**) Interactions specific to the WT are depicted in the WT structure. (**c**) Interactions common to the WT and the mutant are depicted in the mutant structure. (**d**) Interactions specific to the mutant are depicted in the mutant structure. Light (L) chain, heavy (H) chain, and antigen are colored in green, cyan, and magenta, respectively. Interactions are depicted in yellow dashed lines. Water molecules are depicted by red spheres. The interactions of the rest of the DP mutants are given in Figs. S2–4.

The primary process used to select the ten mutants detailed above from among the mutant 3D models was a visual inspection of the interactions between mutated residues, the antigen, and water molecules. After all of the interactions were identified, changes in interactions from the WT D3h44 to the mutant were checked (Fig. 2 and Figs. S2–5).

In the H-K30Q/H-E54H DP mutant (Fig. 2), many interactions in the WT (Fig. 2a) were preserved in the mutant (Fig. 2c). The CH–O and electrostatic interactions between H-K30 and H-E54 of the WT (Fig. 2b) were changed and compensated for by the hydrogen bonds between H-Q30 and H-H54 of the mutant (Fig. 2d). In addition, H-H54 of the mutant forms hydrogen bonds with the S202 of the antigen. For these reasons, the H-K30Q/H-E54H mutant was selected for further experimental evaluation.

In the L-N34D/L-H91S DP mutant (Fig. S2), the L-N34 of the WT interacted with L-H91, L-Y36, L-Y49 via hydrogen bond and CH-O interactions (Fig. S2a,b). The interactions between L-N34 and L-H91 were not estimated to be extremely strong, because the angle of interaction between L-N34 and L-H91 is almost perpendicular, and is not ideal geometry for a hydrogen bond of NH⋯O=C. The interactions between L-N34 and L-H91 are replaced by a hydrogen bond interaction with an ideal geometry between L-D34 and L-S91, accompanied by a CH–O interaction in the L-N34D/L-H91S mutation.

In the H-Y33Q/H-A101R DP mutant (Fig. S3), the interactions between H-Y33 and H-A101 of the WT were mediated by water molecules, and no direct interaction was observed (Fig. S3b). Direct interactions between H-Q33 and H-R101 via two hydrogen bonds could be expected in the H-Y33Q/H-A101R mutant (Fig. S3d).

In the H-A101S/H-A102H DP mutant (Fig. S4), only van der Waals interactions between H-A102 and P194 of the antigen were observed in the WT (Fig. S4b). H-A101S/H-A102H mutation resulted in the attachment of a hydroxyl group to H-A101 and imidazole to H-A102, respectively. These additional functional groups produce favorable interactions, such as CH–O, CH–π, electrostatic and van der Waals interactions between the antigen and the neighboring residues of the antibody (Fig. S4d).

The evaluation of the six SP mutations was carried out in the same way as those of the DP mutations. The interactions in the SP mutants are shown in Fig. S5.

### Affinity improvements

**Figure 3:**
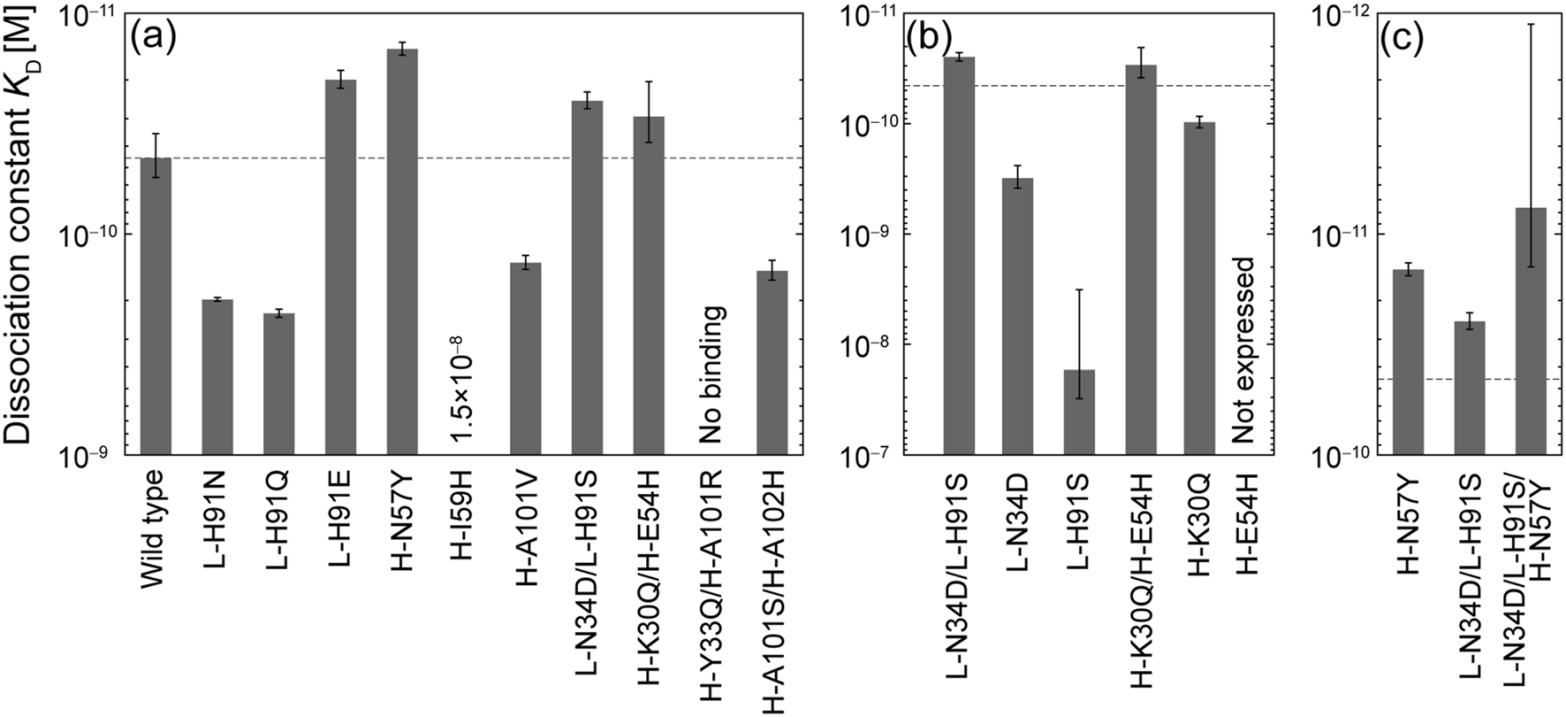
(**a**) Affinity of wild-type (WT) and ten mutants, (**b**) individual single-point (SP) mutants of double-point (DP) mutants, (**c**) triple-point mutant combining two mutations (**c**). Dashed lines indicate the affinity of the WT. Error bars represent the standard deviations of three independent measurements.

The binding affinities of the four DP and six SP mutants to the antigen were measured using surface plasmon resonance (SPR) (See Fig. S6 for sensorgrams). Two out of four DP mutants showed affinity comparable to or better than that of the WT (H-K30Q/H-E54H: *K*_D_ = 29 ± 9 pM, L-N34D/L-H91S: *K*_D_ = 25 ± 2 pM, cf. WT: *K*_D_ = 45 ± 10 pM) (Table 1a and Fig. 3a). The H-A101S/H-A102H DP mutant had a three-fold reduction of affinity (*K*_D_ = 150 ± 15 pM), and the H-Y33Q/H-A101R DP mutant had a complete loss of affinity. Two of the six SP mutants also showed improved affinity (H-N57Y: *K*_D_ = 15 ± 1 pM, L-H91E: *K*_D_ = 20 ± 2 pM). The other four SP mutants exhibited a reduction in affinity ranging from 3.1- to 333-fold.

**Table 1:** Affinity and kinetic parameters determined by surface plasmon resonance (SPR).

Antibody	*k*_on_ (10^6^ M^-1^ s^-1^)		*k*_off_ (10^–6^ s^-1^)		*K*_D_ (pM)		${\text{K}}_{\text{D}}^{\text{ WT}}\text{/}{\text{K}}_{\text{D}}^{\text{ Mut}}$	
	Average	SD	Average	SD	Average	SD		Uncertainty
**(a) Wild-type and mutants (first round)**								
Wild-type	1.4	0.041	62	15	45	10		
L-H91N	1.3	0.098	260	15	200	3.7	0.23	0.05
L-H91Q	1.7	0.12	380	16	230	9.7	0.20	0.05
L-H91E	1.4	0.15	27	1.2	20	1.9	2.3	0.6
H-N57Y	1.3	0.18	18	1.5	15	0.98	3.1	0.7
H-I59H	3.6	2.1	38,000	7900	15,000	10,000	0.0031	0.002
H-A101V	1.3	0.018	180	13	140	9.9	0.33	0.08
L-N34D/L-H91S	1.2	0.20	30	3.3	25	2.2	1.8	0.4
H-K30Q/H-E54H	0.94	0.21	26	1.8	29	9	1.5	0.6
H-Y33Q/H-A101R	No binding							
H-A101S/H-A102H	1.2	0.095	180	11	150	15	0.31	0.08
**(b) Individual single-point mutants**								
L-N34D	2.0	0.25	620	160	310	72	0.14	0.05
L-H91S	16	23	59,000	29,000	17,000	14,000	0.0027	0.002
H-K30Q	1.5	0.24	140	11	97	12	0.46	0.1
H-E54H	Not determined due to very low expression level							
**(c) Triple-point mutant**								
L-N34D/L-H91S/H-N57Y	1.6	0.35	14	12	7.6	6.5	5.9	5.2

After successfully obtaining the DP mutants L-N34D/L-H91S and H-K30Q/H-E54H with improved or comparable affinity, the individual SP mutants L-N34D, L-H91S, H-K30Q, and H-E54H were expressed, and their binding affinities were measured, in order to examine the effect of the individual SP mutations of the DP mutants. As shown in Fig. 3b and Table 1b, the binding affinities of the SP mutants L-N34D and L-H91S decreased to *K*_D_ = 310 pM and *K*_D_ = 17,000 pM, respectively. The binding affinity of the SP mutant H-K30Q decreased to *K*_D_ = 97 pM. The H-E54H mutant was not expressed.

Finally, the triple-point mutant L-N34D/L-H91S/H-N57Y was designed and its binding affinity was examined. The affinity was expected to be increased because the affinities of both L-N34D/L-H91S DP and H-N57Y SP mutants were increased; the locations of L-N34D/L-H91S and H-N57Y are distant in 3D (Fig. 1); and the effects of the mutations are expected to be independent each other and additive. The affinity of the triple-point mutant L-N34D/L-H91S/H-N57Y was increased to 7.6 pM, although the uncertainty was relatively large (± 6.5 pM), because its affinity reached the limit of measurement (Fig. 3c and Table 1c).

### Binding kinetics

**Figure 4:**
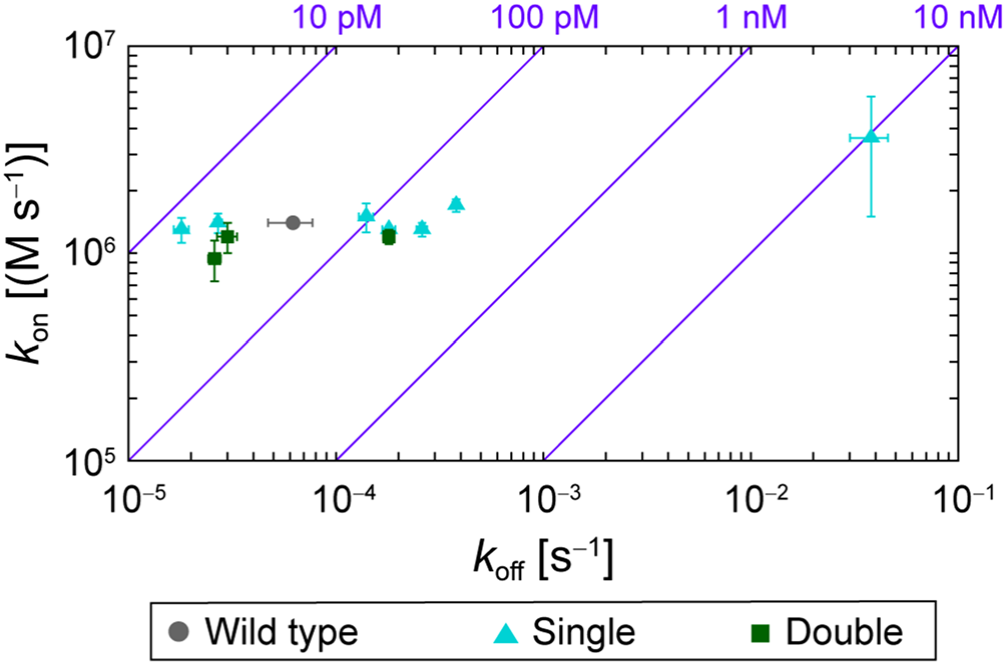
Kinetic parameters of binding measured by surface plasmon resonance. The dissociation and association rate constants, *k*_off_ and *k*_on_, respectively, are plotted with error bars representing the standard deviations of three independent measurements. Purple diagonal lines show the contours of the dissociation constant, *K*_D_ = *k*_off_/*k*_on_. The data are presented in Table 1.

The effect of the mutations on the kinetic parameters of binding was examined. The change in affinity of the four DP mutants and six SP mutants was mainly governed by a change in dissociation rates *k*_off_ when *K*_D_ < 1 nM (Fig. 4). This observation was true for the individual SP mutants (H-K30Q, L-N34D, and L-H91S) and the triple-point mutant (Fig. S7).

### Stability changes

**Figure 5:**
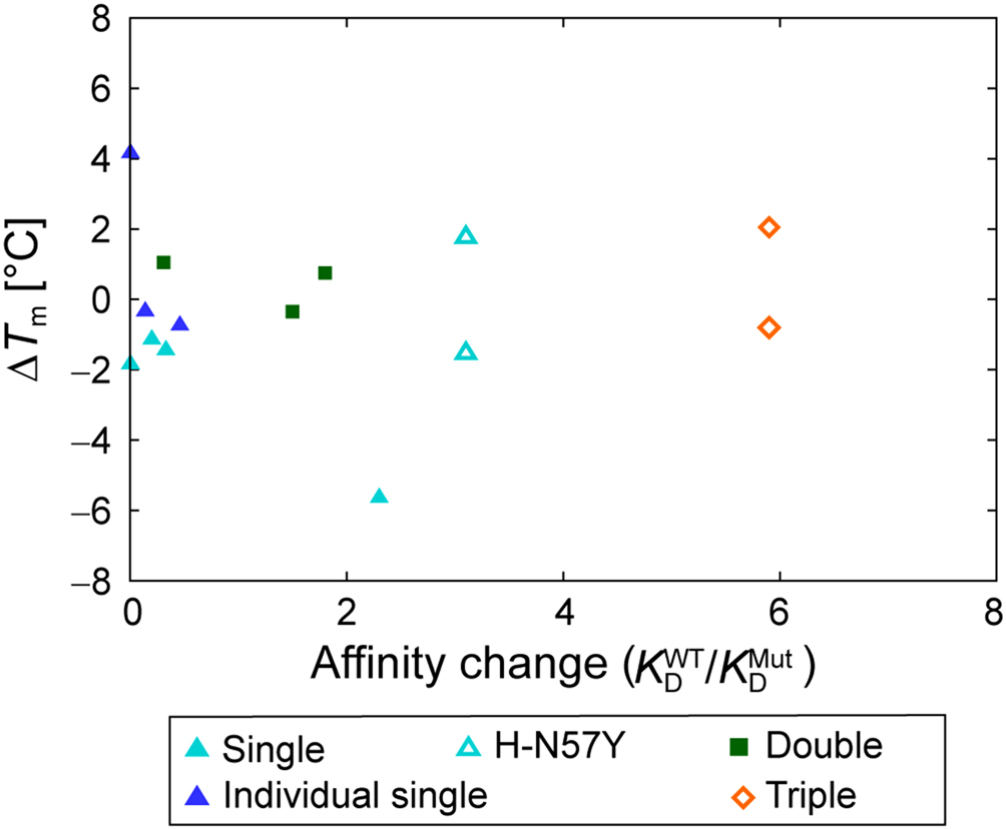
Change in melting temperature, ∆*T*_m_, of antibodies upon mutation, as measured by DSC. The data are presented in Table S1. The mutant showing double melting peaks is designated by an unfilled symbol. H-Y33Q/H-A101R is not shown, because it lost binding affinity.

Changes in the stability of the mutants were assessed by comparing the melting temperature change ∆*T*_m_ with that of the WT. *T*_m_ was measured using differential scanning calorimetry (DSC), and the ∆*T*_m_ values of all mutants are listed in Table S1. The maximum increase in the change in melting temperature, ∆*T*_m_, among the 13 mutants was observed to be 4.2 °C, for the individual SP mutant L-H91S derived from the DP mutant, and the maximum decrease, of ∆*T*_m_, was − 5.7 °C for the SP mutant, L-H91E from the first-round design. The ∆*T*_m_ values of 8 out of 11 mutants, excluding the H-N57Y SP mutation, which exhibited two ∆*T*_m_ values, were within ± 2 °C. No correlation between the change of the stability and the affinity change was observed for the ten mutants from the first-round design (Fig. 5). The mutants that showed fold-affinity improvements of > 1 were as stable as the WT, except for the L-H91E mutant and those involving H-N57Y. The reason H-N57Y showed two melting temperatures (Fig. S8) is unclear.

## Discussion

The successful identification of two DP mutants, H-K30Q/H-E54H and L-N34D/L-H91S with affinity improved over or comparable to that of the WT D3h44 demonstrates the success of the double-point mutation strategy. The key process for selecting the four DP mutants from the 3D models of 59 sets of residue pairs was a visual inspection of the change in interactions between the WT and the DP mutant. The successful acquisition of two DP mutants also indicated that the analysis of the interactions between the mutated residues, antibody residues close to the mutated residues, the antigen, and surrounding water molecules is informative and useful for the design of novel cooperative DP mutants. This work also indicates that it may be possible to predict the binding affinity of designed antibodies based on information about their interactions.

**Figure 6:**
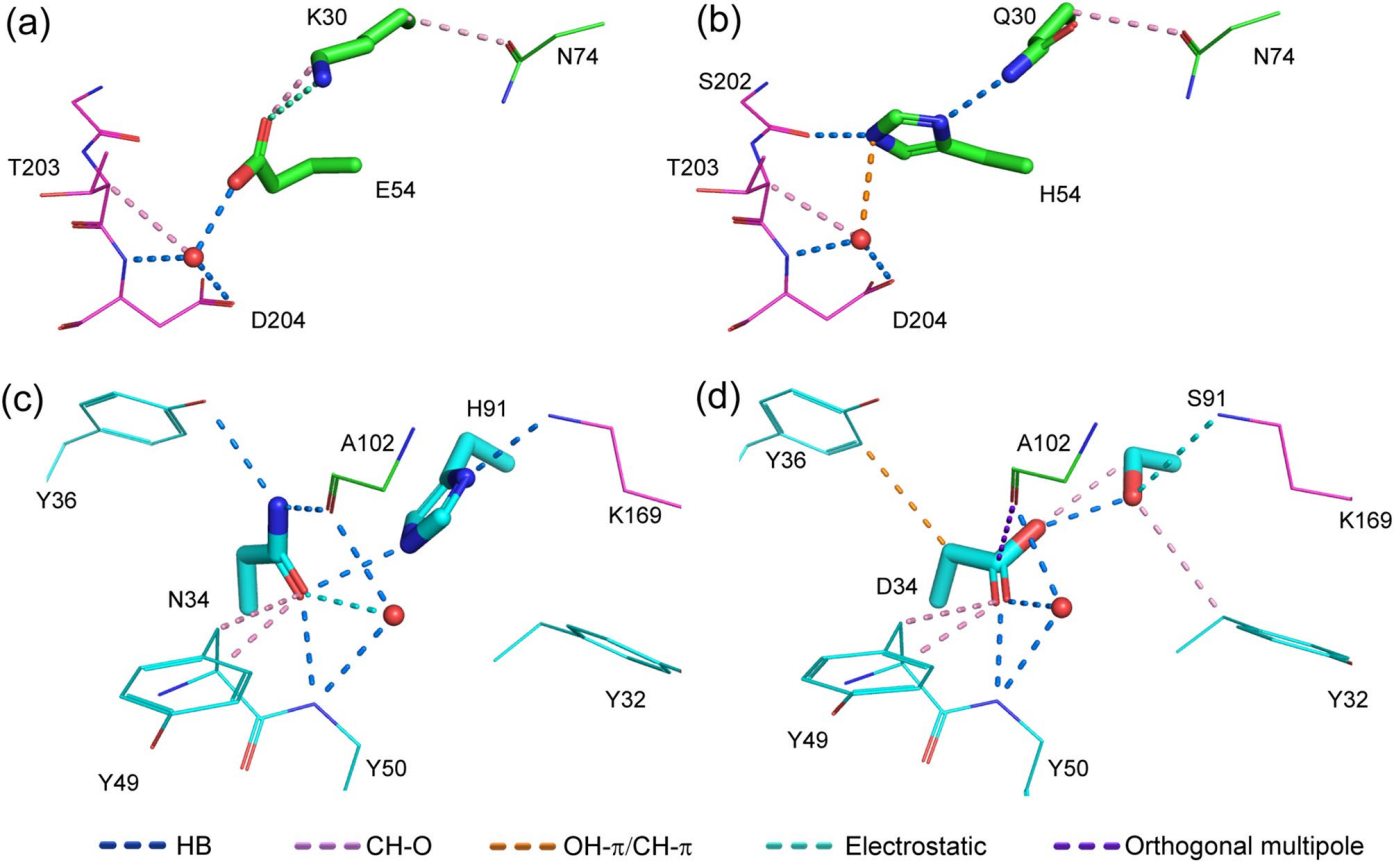
(**a**) Interactions of H-K30 and H-E54 of the wild-type (WT). (**b**) Interactions of H-K30Q and H-E54H. (**c**) Interactions of L-N34 and L-H91 of the WT. (**d**) Interactions of L-N34D and L-H91S. The antigen and the heavy chain of the antibody are colored in magenta and green, respectively. A water molecule is depicted as a red sphere.

The H-K30Q/H-E54H DP mutant exhibited a 1.5-fold increase in affinity (29 pM) over that of the WT D3h44 (45 pM). There are two possible reasons for the improvement in affinity of the DP mutant. One is the reinforcement of the interaction between the antigen and the antibody by the additional hydrogen bond between H-H54 and S202 of the antigen (Fig. 6b). The other is the replacement of weaker electrostatic and CH-O interactions between H-K30 and H-E54 of the WT (Fig. 6a) by the strong hydrogen bond between H-Q30 and H-H54 of the DP mutant (Fig. 6b). The interaction between H-K30 and H-E54 is not a strong ionic interaction, but a less strong electrostatic interaction, based on their distance and angle. These preferable interactions between the antigen and the DP mutant, and between the mutated residues, are a good example of the cooperative mutation produced by the double-point mutation strategy combined with interaction analysis.

The other successful DP mutant was L-N34D/L-H91S, with a 1.8-fold increase in affinity (25 pM) over that of the WT (45 pM). This case is informative, because the DP mutation including residue 34, which does not contact with the antigen, improved the binding affinity. The increase in affinity may have occurred because the mutation provided additional weak interactions, such as CH–O, while preserving and/or replacing the interactions around L-34 and L-91. Two new CH-O interactions were formed: between L-D34 and L-S91 and between L-S91 and L-Y32 (Fig. 6d). Although a CH–O interaction is a weak interaction, two additional CH–O interactions contributed to the stabilization of the whole antibody/antigen complex and increased the affinity of the DP mutant (Fig. 6d). With respect to the interactions between the antigen and the mutated residues, the interaction networks of the WT/antigen complex (K169 of antigen, L-H91 and L-N34) were successfully replaced by the interaction networks (K169 of antigen, L-S91 and L-D34) of the DP mutant/antigen (Fig. 6c,d). Overall, a 1.8-fold increase in the affinity of the L-N34D/L-H91S DP mutant appeared to be achieved. This is another example of the cooperative mutations produced by the “DP mutation” strategy with the interaction analysis.

One of the unique features of the interaction analysis is the inclusion of weak interactions such as CH–O and CH–π. These interactions are recognized as important for the structure-based drug design of small molecules^19,20^ as described in “Introduction”. In general, these weak interactions require small desolvation energy. On the contrary, high desolvation energy is required to form a strong hydrogen bond interaction. The balance between gain (formation of interaction) and loss (desolvation) of these weak interactions have attracted attention. In the case of this study, such weak interactions were frequently observed. For example, 14 and 16 CH–O interactions between mutated residues and their surrounding residues were found in the WT and L-N34D/L-H91S DP mutant, respectively. These numbers indicate that the effect of these weak interactions is not negligible, even though the individual interaction is weak.

Although two DP mutants with affinity improved over or comparable to the WT were successfully obtained, the current procedure for the selection of DP mutants is not perfect, because not all of the four DP mutants showed good affinity. For the H-A101S/H-A102H DP mutant, the affinity decreased to 150 pM, a 3.3-fold reduction compared to the WT. This reduction was probably due to the size of the mutated residues, especially H-H102. Based on the H-A101S/H-A102H mutant model, it was expected that mutations would increase favorable interactions, as described in the “Results” section. However, the mutation from the smaller H-A102 to the larger H-H102 triggers conformational changes of I152 of the antigen and L-Y49, by van der Waals collisions (Fig. S9). The instability stemming from the conformational changes may cause the 3.3-fold reduction of the affinity.

The H-Y33Q/H-A101R DP mutant showed complete loss of affinity. The main reason for the loss of the affinity appears to arise from the conformational preference of the mutated residues. The conformational preference of H-Q33 of the model is 3.4% of all of 54 conformations of Gln and that of the H-R101 model is 0.06% of all of 81 side chain conformations of Arg, according to the rotamer library of amino acids^28^ (Fig. S10 and Table S3). Thus, the preferable interactions between H-Q33 and H-R101 observed in the model would rarely occur; the probability is estimated at 3.4% × 0.06% = 0.002%.

The other key finding was that cooperative DP mutants could not be obtained when a typical procedure, the combination of improved SP mutants, was taken. In the case of the H-K30Q/H-E54H cooperative DP mutant (*K*_D_ = 29 pM), the individual SP mutant H-K30Q showed a 2.2-fold reduction in affinity (*K*_D_ = 97 pM) compared with the WT (*K*_D_ = 45 pM), and the H-E54H SP mutant was not expressed. Using this approach, the chance to examine the affinity of the mutant with a combination of the residue with a 2.2-fold reduction of the affinity (H-K30Q) and the residue not expressed (H-E54H) is rare. In the L-N34D/L-H91S DP mutant (*K*_D_ = 25 pM), the individual SP mutant L-N34D exhibited a 6.9-fold reduction in affinity (*K*_D_ = 310 pM) and the affinity of the L-H91S SP mutant was observed to be 17,000 pM, 1/378 of that of the WT. The probability of developing a mutant with the combination of a residue with a 6.9-fold reduction (L-N34D) and a residue with a 378-fold reduction (L-H91S) is very low. A combination of the negative effects of an SP mutation—a reduction of affinity or no expression—and another SP mutation can result in a positive effect: an increase in affinity. Simultaneous cooperative mutations arising from the complementarity of two residues are thought to be important to this phenomenon.

Because of resource limitations, the number of mutants selected for experimental evaluation in the first round of the design was set to ten. Despite the generation of 3D model structures for 59 sets of residue pairs with 19 amino acids for each residue position, four DP mutants were selected, because they were the only DP mutants that had passed visual inspection of interactions. This low number suggested that the simultaneous mutation of two contacting residues with favorable interactions might be rare.

Another possibility is that not all of the potentially active mutants with favorable interactions had been pursued. In the evaluation of the mutant models, visual inspection was carried out on only the top 20 of 361 DP mutant models (every pair set of two amino acid positions, 19 × 19 = 361), based on the MM/GBVI binding energy value calculated using MOE^29^. The other mutant models were discarded. The number of models to be visually inspected was increased to a maximum of 80 if a mutant with favorable interactions did not exist among the top 20 models. Neither the total nor the electrostatic^12^ MM/GBVI binding energy correlated with the change in experimental binding affinity (Fig. S11). Thus, the filtering of the mutant models according to MM/GBVI binding energy is not ideal, and there is a possibility that not all of the DP mutants with favorable interactions were subjected to further visual inspection. Although not ideal, filtering by MM/GBVI binding energy followed by visual inspection of the interactions appeared to be effective, given that the first round of design led to the identification of two DP mutants with improved or comparable affinity to that of the WT.

The affinity of the triple-point mutant L-N34D/L-H91S/H-N57Y was increased to 7.6 pM, a 5.9-, 3.2- and 2.0-fold increase over the affinity of the WT, L-N34D/L-H91S DP mutant, and H-N57Y SP mutant, respectively. The triple-point mutant was created to add the positive effects of two distantly located mutants, L-N34D/L-H91S and H-N57Y, which are 17 Å apart in 3D (Fig. 1 and Fig. S12). The 5.9-fold increase in affinity of the triple-point mutant was nearly equal to the product of the fold increase of the N34D/L-H91S DP mutant, 3.2, and that of H-N57Y SP mutant, 2.0. In this case, the assumption that the effect of the mutations of distant locations in 3D are independent each other and could be additive was validated. Although the starting point of the affinity of the WT was very high (45 pM), a 5.9-fold increase of the affinity of the triple-point mutant (7.6 pM) was achieved. This achievement reinforced the importance of 3D information in the design of mutants, because the addition of the positive effect of two mutants is thought to be possible only when the mutated residues are distant in 3D.

The change in the binding affinity, *K*_D_, is primarily governed by the change of the dissociation constant *k*_off_. In the design process, the 3D structures of the antigen/antibody complex were examined with the focus on short-range interactions around the mutation residues. It has been reported that the dissociation process is governed by short-range interactions between proteins^30^. This may be the possible explanation of our results on *k*_off_. In addition, the affinity changes derived from structure-based antibody design based on SP mutations are typically governed by change in *k*_off_^16^. The design of mutants based on the 3D structure of the antigen/antibody complex may not produce a significant effect on the association rate, *k*_on_. Design based on the complex, and which is intended to stabilize the complex, did affect the dissociation rate, *k*_off_. Thus, *k*_off_ was improved if the design was successful and deteriorated if failed.

The stability of the mutants was not explicitly considered in the design process in the same way as *k*_on_ and *k*_off_. Although some studies have reported that affinity and stability have a trade-off relationship^31^, the change of the melting temperature ∆*T*_m_ fell within the range of − 2 to + 2 °C for most of our mutants (Fig. 5). No correlation between the affinity change and ∆*T*_m_ was observed. The lack of correlation between the affinity and stability changes may indicate that the consideration of the preservation of the intra-antibody interactions in the design process contributed to preventing the mutants from significant reduction in stability.

Ten DP and SP mutants were prospectively designed using a double-point mutation strategy, with interaction analysis based on the 3D structure of the mutant models. Two DP and two SP mutants with an affinity improved over or comparable to that of the WT were successfully obtained. Of the four individual SP mutants, which involved the constituent residues of the two active DP mutants, three showed decreased affinity, and one was not expressed. These results indicated that the two active DP mutants are hard to be obtained using a SP mutation strategy. The triple-point mutant, which is a combination of the active DP and SP mutants, was designed and a further increase in affinity was achieved.

Although the DP mutation strategy with interaction analysis was successful, there are several issues still to be overcome. The most critical issue is the manual identification of the interactions, which is labor-intensive and time-consuming. Automating the identification of interactions based on the 3D structure of the mutation model is a current focus of our research, and the software for automatic identification of interactions has been prototyped. As preliminary test cases, this prototype software was applied to the ten mutants designed, and it reproduced the interactions obtained manually. The details of the automatic identification of interactions will be published elsewhere. Another important issue is the best way to evaluate the changes of interactions introduced by mutation. Currently the evaluation is not quantitative and is based on personal experience of the structure-based design. Further investigation of the relationships between the changes in affinity and changes of the interactions, based on data provided by the automatic interaction detection software and the automatic mutant modeling software, is required. Another issue is the modeling of the mutant/antigen complex including water molecules. Regarding the relationship between water rearrangement in the mutant models and affinity change, any significant relation between the extent of water rearrangement and the fold improvement in affinity was not observed (Fig. S13). Affinity improvement occurred in both cases where the water rearrangement is small (RMSD < 0.2 Å) and relatively large (RMSD > 0.6 Å). These observations indicate that the water rearrangement of the mutant models did not affect the results largely in our study. However, this may not be true in all cases. As an example of water rearrangement, it was found that a cavity produced by the mutation, which may accommodate a water molecule, was not fulfilled in some mutant models when all possible combinations of DP mutants were examined. Thus, the modeling of DP mutants involving water molecules is currently under consideration. For example, a short molecular dynamics simulation of solvated antigen/antibody complex with restraining protein atoms is an option for realizing water rearrangement in an appropriate manner. In addition, free-energy perturbation of DP and multiple mutants for affinity prediction is thought to be one of the directions for improving the accuracy of the affinity prediction^32^. Though we assumed that the structure of the overall antigen–antibody complex was retained upon mutation^33^, there might be the cases where DP and even SP mutation induces a large structural change, which is outside of the scope of the mutant modeling method we adopted in this study. Other methods may be necessary to study such a scenario. The inclusion of the effect of side chain flexibility is another issue, as seen in the failure of the design of the H-Y33Q/H-A101R DP mutant. After resolving the issues described above, we hope to extend the DP mutation strategy to a triple-point mutation strategy.

## Methods

### Selection of mutations

**Figure 7:**
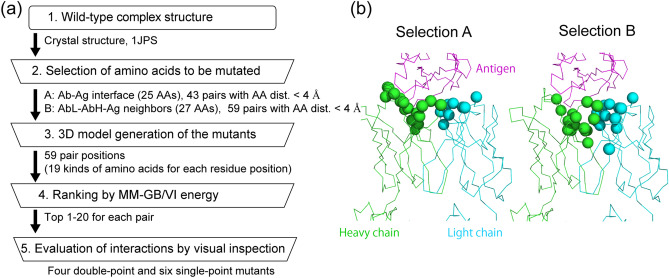
(**a**) Workflow for selecting mutants for experimental evaluation of affinity. Specific values obtained in this study are provided outside the boxes. (**b**) Cα atoms of amino acids examined in Step 2 are depicted as spheres.

The mutants were designed based on the workflow describe below and shown in Fig. 7a.

(Step 1) The anti-TF antibody D3h44 was used as a target. The crystal structure of D3h44 Fab/TF was retrieved from the protein data bank^34^ (PDB ID: 1JPS^22^).

(Step 2) Using the crystal structure 1JPS, the residues in the antibody that contacts the antigen at a distance of < 4 Å (Fig. 7b, Selection A), and the residues in the chain L (or H) that contacts with both the chain H (or L) and the antigen at a distance of < 6 Å (Fig. 7b, Selection B) were selected as candidate residues to be mutated. Selection B was prepared with the intent of including the residues of the antibody not in direct contact with the antigen, but adjacent to the residues of the antibody in contact with the antigen. For each selection, residue pairs whose distance was within 4 Å, were selected as candidates for DP mutations and subjected to Step 3.

(Step 3) The hydrogens were added to the crystal structure 1JPS using the Protonate3D method^35^ of MOE^29^^.^ After applying the ff14SB force field^36^, the structure was minimized to the root mean square gradient criterion of 0.1 kcal/(mol Å) over atoms. Before exhaustive modeling of the DP mutants, a 3D model of the WT for each DP mutation pair was prepared and compared with the crystallographic structure, in order to check the accuracy and the feasibility of the mutant modeling for each pair position. For example, when the H-K30/H-E54 DP mutation was tried, the 3D model of H-K30K/H-E54E mutant (= WT) was constructed using the procedure used for making the mutant model described below. The WT models were evaluated by the RMSD between the atoms within 4.5 Å from of the residues to be mutated of the WT model and the corresponding atoms of the crystal structure. We assumed that the introduction of SP or DP mutations would not produce a significant change in structure and binding mode of the antibody and antigen complex^33^. Thus, the WT models with an RMSD exceeding 0.5 Å were excluded from further modeling of the DP mutants, because a large RMSD indicates greater difficulty in building a 3D model under the modelling method employed. After RMSD filtering, 59 sets of the residue pairs for Selections A and B were selected. For each set, the 3D mutant models of all possible combinations, 19 (natural amino acids, excluding Cys) × 19 = 361, were automatically built using MOE software. The modelling procedure of the MOE protein design residue scan module^29^ is as follows: when the DP mutants of each residue pair is examined, both residues of the WT to be mutated are simultaneously mutated to one of 19 amino acids. During mutant modelling, any water molecules other than crystallographic water were not added or removed. After conformational search by unary quadratic optimization (UQO^35^), one selected structure is further minimized. During the minimization, water molecules are allowed to move.

(Step 4) In order to select the mutant/antibody models for the visual inspection of the interactions, the models were ranked by affinity score. The affinity score was calculated, using MOE software, as the difference of MM/GBVI energies^27^ between the complex, antibody and antigen. For each set, the top 20 models with a good affinity scores were subjected to visual inspection. The number of the models to be visually inspected was increased to a maximum of 80 if a mutant with favorable interactions did not exist among top 20 models.

(Step 5) In the visual inspection of the mutant/antigen complex models, all of the interactions, including weak interactions, were identified by measuring the geometry, such as the distances and angles between atoms using the PyMOL software^37^. The interactions considered are listed in Table S2^23–26,38–41^. An interaction was defined as existing only when the distance between heavy atoms was within 4 Å. Finally, four DP and six SP mutants among the models of 59 sets of the residue pairs were selected for further experimental evaluation.

### Expression and purification of 1JPS Fab and TF

The sequence of anti-TF Fab (1JPS) was subcloned into a pCDNA3.4 topo vector (Thermo) with a C-terminal myc-six histidine tag (EQKLISEEDLNSAVDHHHHHH). 1JPS mutants were prepared by site-directed mutagenesis PCR using KOD-mutagenesis kits (TOYOBO). The protocol was slightly modified, using KOD-ONE polymerase (TOYOBO) instead of KOD-plus. 1JPS Fab and mutants were expressed using the ExpiCHO Expression System (Thermo) and followed the max titer protocol. Supernatants of ExpiCHO culture medium were harvested 10–14 days after transfection. Supernatants were dialyzed with 500 mM NaCl and 20 mM Tris–HCl pH 8.0 5 mM imidazole for overnight, after separation of cells by brief centrifugation and filtration. Supernatants were loaded to 1 ml column volume Ni–NTA resin (QIAGEN), and washed with 10 ml 500 mM NaCl, 20 mM Tris–HCl pH 8.0, 20 mM Imidazole buffer. Fab was eluted with 5 ml 500 mM NaCl, 20 mM Tris–HCl pH 8.0, 200 mM Imidazole buffer. Eluted Fab was dialyzed with PBS and purified by size exclusion chromatography using a HiLoad 16/60 Superdex 200 pg column (GE Healthcare).

The gene encoding TF (S33 to E251) was subcloned into pET-28b (+) with an N-terminal six histidine-SUMO-tag. pET28-6HisSUMOTF was expressed using the BL21 (DE3) expression system with LB medium. Briefly, BL21 was cultured at 37 °C until the O.D. reached 0.8, and protein expression was induced using isopropyl β-d-1-thiogalactopyranoside (final concentration, 500 μM) followed by incubation for 18 to 20 h at 20 °C. Pellets of *E. coli* cells were resuspended in 500 mM NaCl, 5 mM imidazole, 20 mM Tris–HCl pH 8.0 5 mM imidazole. The cells were disrupted by sonication and centrifuged at 40,000*g* for 30 min. Supernatants were loaded to 1 ml column volume Ni-NTA resin (QIAGEN), the column was washed with 10 ml 500 mM NaCl, 20 mM Tris–HCl pH 8.0, 20 mM imidazole buffer, and eluted with 5 ml 500 mM NaCl, 20 mM Tris–HCl pH 8.0, 200 mM imidazole buffer. To remove the SUMO-tag, eluted TF was incubated with the Ulp1 enzyme with an N-terminal six histidine tag, which cleaves the 6xHis-SUMO-tag in dialysis membrane with 20 mM Tris, 500 mM NaCl, 5 mM imidazole for over 12 h. Cleaved 6xHis-SUMO-tag and 6xHis-Ulp-1 were removed using a Ni-affinity column, and flow-through TF samples were collected and dialyzed in PBS. Tag-free TF was purified by size exclusion chromatography using a HiLoad 16/60 Superdex 75 pg column (GE Healthcare). The purity of all proteins was analyzed by sodium dodecyl sulfate polyacrylamide gel electrophoresis followed by staining with Coomassie brilliant blue (Fig. S14).

### SPR analysis using Biacore 8K

In the report by Presta et al.^21^, the interaction between D3h44 (1JPS WT)^22^ and TF was analyzed using Biacore 2000. We optimized the experimental conditions and re-evaluated the kinetic parameters because we used an upgraded instrument, Biacore 8K. The kinetic parameters were almost identical to those described in the report. We optimized the experimental conditions further, e.g. level of immobilized TF on the sensor chip, analyte concentration of the Fab, association time, and dissociation time, in order to obtain the best fitting curve for the most reliable data fitting and kinetic parameters.

The purified TF was dialyzed in PBS supplemented with 0.005% Tween-20. The TF was immobilized on a CM5 sensor chip (GE Healthcare) by means of an amine coupling method with an immobilization target of 450–750 response units. Interactions of antibodies with TF were analyzed using a single-cycle kinetics protocol with an association time of 120 s and a dissociation time of 1800 s. No regeneration of the sensor chips was performed. Data were analyzed using Biacore Insight evaluation software Version2.0.15.12933^42^. The measurements were repeated three times, and average association (*k*_on_) and dissociation (*k*_off_) rate constants, dissociation constant (*K*_D_), and their standard deviations (SD) were determined. The uncertainty of ${\text{K}}_{{\text{D}}}^{{\text{ WT}}} {\text{/K}}_{{\text{D}}}^{{\text{ Mut}}}$ was calculated from ${\text{K}}_{{\text{D}}}^{{\text{ WT}}}$ and ${\text{K}}_{{\text{D}}}^{{\text{ Mut}}}$ and their SDs via the rule of error propagation^43^.

### DSC

Thermal stability was evaluated using an automated MicroCal PEAQ-DSC (Malvern Panalytical). D3h44 (1JPS WT) and the mutants were dialyzed against PBS and concentrated using Amicon Ultra-4 30K (UFC 803024, Merck, Millipore). Twenty-five μM of each Fab sample was loaded onto a 96-well plate and set in the sample compartment at 10 °C. Each sample–buffer pair was scanned over a range of 20–110 °C at a rate of 1 °C/min in PBS buffer. Data were analyzed using MicroCal PEAQ-DSC software version 1.40^44,45^, and peak integration was performed using a non-2-state model.

## Supplementary information

Supplementary Information.
