# Author Correction: Structure-based design and discovery of novel anti-tissue factor antibodies with cooperative double-point mutations, using interaction analysis

**DOI:** 10.1038/s41598-021-97892-2

**Published:** 2021-09-07

**Authors:** Shuntaro Chiba, Aki Tanabe, Makoto Nakakido, Yasushi Okuno, Kouhei Tsumoto, Masateru Ohta

**Affiliations:** 1 Medical Sciences Innovation Hub Program, RIKEN, 1-7-22, Suehiro-cho, Tsurumi-ku, Yokohama, 230-0045 Japan; 2 Department of Bioengineering, School of Engineering, The University of Tokyo, Bunkyo-ku, Tokyo, 113-8656 Japan; 3 Graduate School of Medicine, Kyoto University, Sakyo-ku, Kyoto, 606-8507 Japan; 4 The Institute of Medical Science, The University of Tokyo, Minato-ku, Tokyo, 108-8639 Japan

Correction to *Scientific Reports* 10.1038/s41598-020-74545-4, published online 16 October 2020

The original version of this Article contained an error in Figure 3, where the labels in panel (b) were permutated. The original Figure 3 and accompanying legend appear below.

The original Article has been corrected.

**Figure 3:**
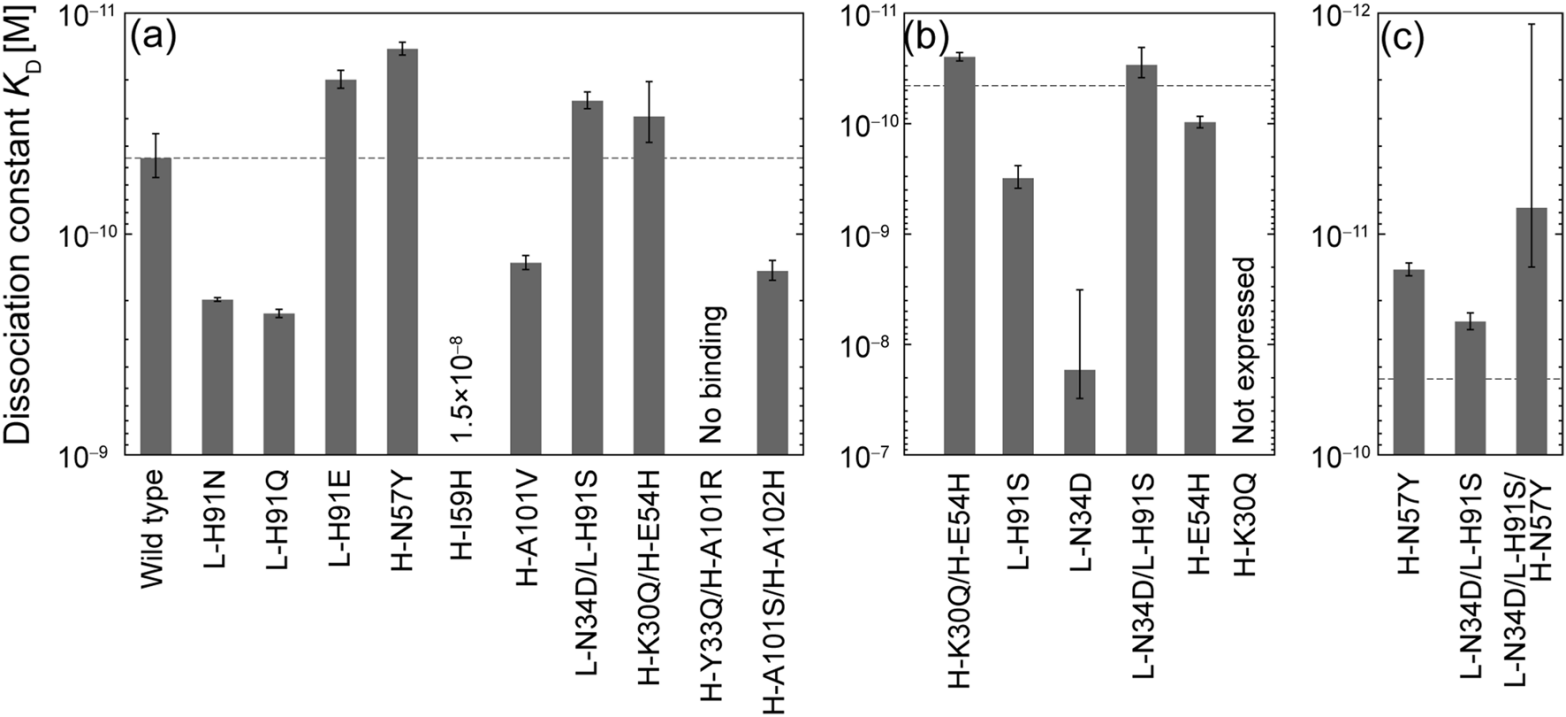
(**a**) Affinity of wild-type (WT) and ten mutants, (**b**) individual single-point (SP) mutants of double-point (DP) mutants, (**c**) triple-point mutant combining two mutations (**c**). Dashed lines indicate the affinity of the WT. Error bars represent the standard deviations of three independent measurements.

