# Supplementary material for: Structure-based design and discovery of novel anti-tissue factor antibodies with cooperative double-point mutations, using interaction analysis
Source: Sci Rep. 2020 Oct 16;10:17590. doi: 10.1038/s41598-020-74545-4 (PMC7567794; doi:10.1038/s41598-020-74545-4)
Supplement: Supplementary file 1 — Supplementary Information. [file 41598_2020_74545_MOESM1_ESM.pdf]

Supporting Information for

Structure-based design and discovery of novel anti-tissue factor antibodies with cooperative double-point mutations, using interaction analysis

Shuntaro Chiba[1], Aki Tanabe[2], Makoto Nakakido[2], Yasushi Okuno[1,3], Kouhei Tsumoto[2,4], Masateru Ohta[1]*

[1]*Medical Sciences Innovation Hub Program, RIKEN, Tsurumi-ku, Yokohama, 230-0045, Japan*

[2]*Department of Bioengineering, School of Engineering, The University of Tokyo, Bunkyo-ku, Tokyo, 113-8656, Japan*

[3]*Graduate School of Medicine, Kyoto University, Sakyo-ku, Kyoto, 606-8507, Japan*

[4]*The Institute of Medical Science, The University of Tokyo, Minato-ku, Tokyo, 108-8639, Japan*

*Corresponding Author:

Masateru Ohta

Medical Sciences Innovation Hub Program, RIKEN

1-7-22, Suehiro-cho, Tsurumi-ku, Yokohama, 230-0045, Japan

masateru.ota@riken.jp

+81-45-503-9644

Table S1. Melting temperature (T_m) of the studied antibodies, determined using differential scanning calorimetry (DSC).

Antibody	T_m (°C)			ΔT_m
	1st test	2nd test [a]	Average	
Wild type	75.8	75.7	75.8	
L-H91N	Not determined due to low expression level			
L-H91Q	74.6		74.6	-1.2
L-H91E	70.1	70.0	70.1	-5.7
H-N57Y[b]	70.9	70.9	70.9	-4.8
	77.5	77.5	77.5	1.8
H-I59H	73.9		73.9	-1.8
H-A101V	74.3		74.3	-1.5
L-N34D/L-H91S	76.5	76.5	76.5	0.8
H-K30Q/H-E54H	75.5	75.3	75.4	-0.3
H-Y33Q/H-A101R	77.9		77.9	2.2
H-A101S/H-A102H	76.8	76.8	76.8	1.1
L-N34D	75.4	75.4	75.4	-0.3
L-H91S	79.9	79.9	79.9	4.2
H-K30Q	75.0	75.0	75.0	-0.8
H-E54H	Not determined due to very low expression level			
H-N57Y/L-N34D/	71.8	71.6	71.7	-4.1
L-H91S[b]	78.1	77.5	77.8	2.1

a) For these mutants, the DSC measurement was repeated twice.

b) Two melting transitions were observed in the mutants with the H-N57Y mutation, as shown in Figure S6.

c) The DSC measurement was conducted for constituent single-point mutants of H-K30Q/H-E54H and L-N34D/L-H91S.

Table S2. Interactions considered during visual inspection.

Interaction	Description	Reference
Hydrogen bond	X[N, O](δ^-) - H(δ^+) … O(δ^-)	
	X[N, O](δ^-) - H(δ^+) … N(δ^-)	
Electrostatic	X[N, O](δ^-) - H(δ^+) … O(δ^-)	
	X[N, O](δ^-) - H(δ^+) … N(δ^-)	
van der Waals	Y_i … Y_j	
CH-O	C(δ^-) - H(δ^+) … O(δ^-)	1
CH-N	C(δ^-) - H(δ^+) … N(δ^-)	1
CH-π	C(δ^-) - H(δ^+) ⊥ π(δ^-)	2
OH-π	O (δ^-) - H(δ^+) ⊥ π(δ^-)	3
NH-π	N(δ^-) - H(δ^+) ⊥ π(δ^-)	4
π–π stacking	π // π	5
S-O	S … O	6
S-π	S ⊥ π	7
Bond Dipole	dipole(δ^+ δ^-) // dipole(δ^- δ^+)	
Orthogonal multipole	dipole(δ^+ δ^-) ⊥ dipole(δ^+ δ^-)	8

δ^+: Positive partial charge, δ^-: Negative partial charge, X: Any heavy elements, X[N,O]: N or O, π: π system, …: Non-bonding interaction, //: parallel interaction, ⊥: Orthogonal interaction. Hydrogen bond and electrostatic interactions were defined as the distance between X - O or N of ≤ 3.2 Å and from 3.2 to 4 Å, respectively. The other interactions were defined if the distance between interacting heavy atoms is < 4 Å.

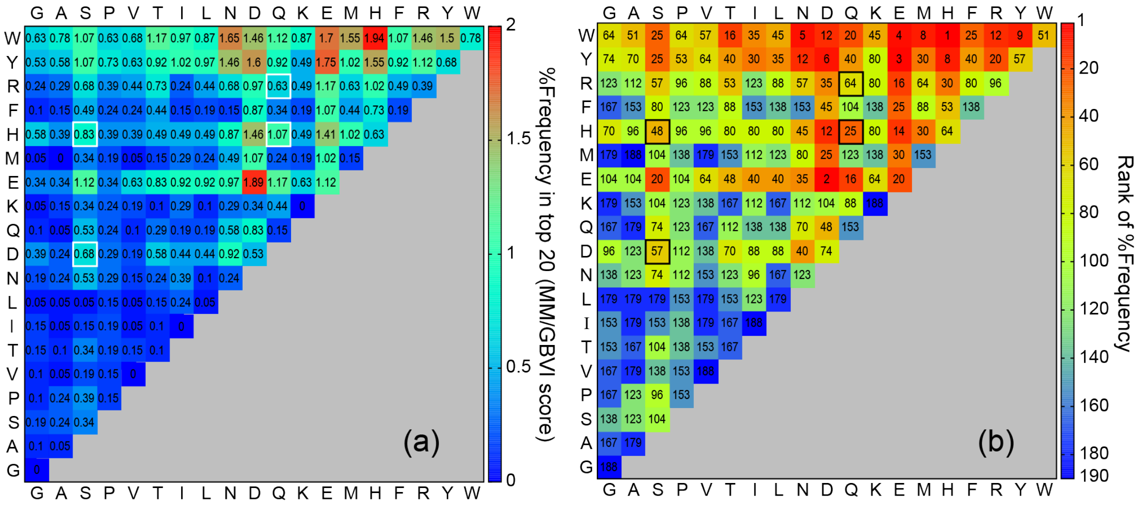

Figure S1. (a) %Frequency of top 20 combinations of amino acids ranked by their molecular mechanics/generalized Born volume integration (MM/GBVI) energy score[9] in the total mutated pairs of 19×19 including the wild-type residues. The average %Frequency was 0.53 ± 0.44%. (b) The rank of %Frequency for each combination. The amino acid combinations of the double-point mutations selected for further experimental evaluation are enclosed in white or black squares in (a) and (b) respectively.

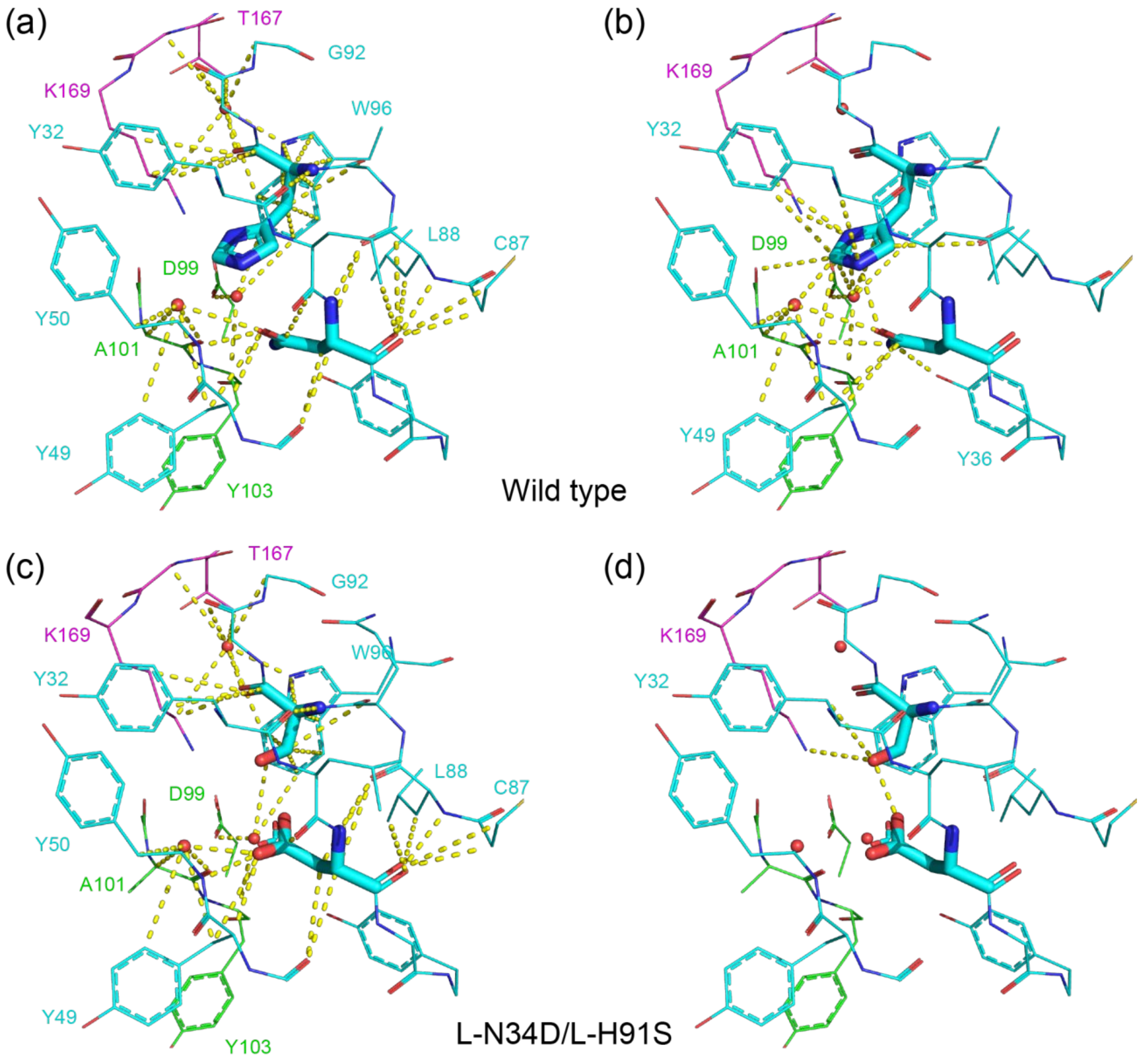

Figure S2. Interactions in the wild-type (WT; PDB ID: 1JPS[10]) and the double-point mutant model (L-N34D/L-H91S). (a) Interactions common to the WT and the mutant are depicted in the WT structure. (b) Interactions specific to the WT are depicted in the WT structure. (c) Interactions common to the WT and the mutant are depicted in the mutant structure. (d) Interactions specific to the mutant are depicted in the mutant structure. Light (L) chain, heavy (H) chain, and antigen are colored in green, cyan, and magenta, respectively. Interactions are depicted as yellow dashed lines. Water molecules are depicted as red spheres.

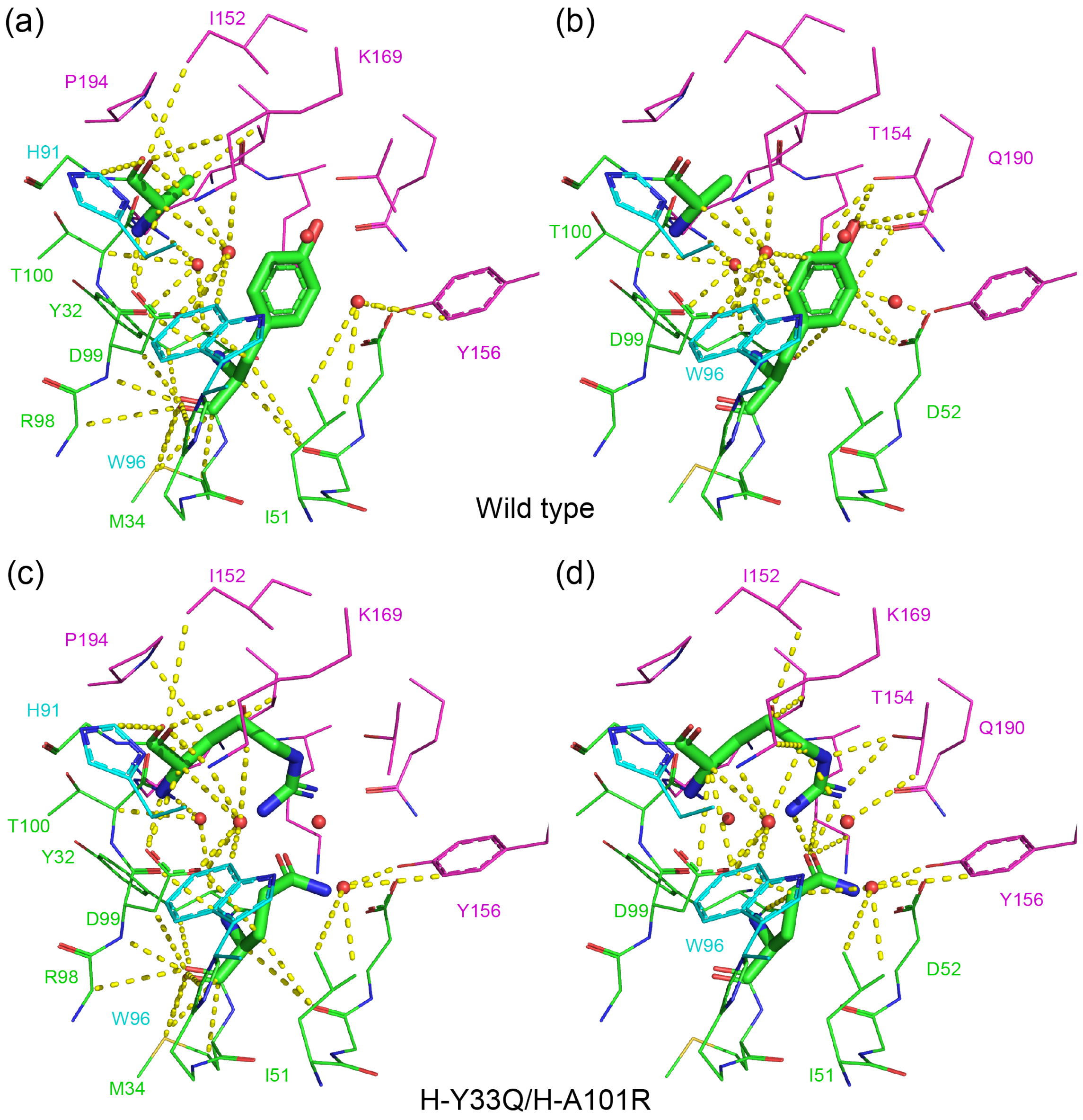

Figure S3. Interactions in the wild-type (WT; PDB ID: 1JPS[10]) and the double-point mutant model (H-Y33Q/H-A101R). (a) Interactions common to the WT and the mutant are depicted in the WT structure. (b) Interactions specific to the WT are depicted in the WT structure. (c) Interactions common to the WT and the mutant are depicted in the mutant structure. (d) Interactions specific to the mutant are depicted in the mutant structure. Light (L) chain, heavy (H) chain, and antigen are colored in green, cyan, and magenta, respectively. Interactions are depicted as yellow dashed lines. Water molecules are depicted as red spheres.

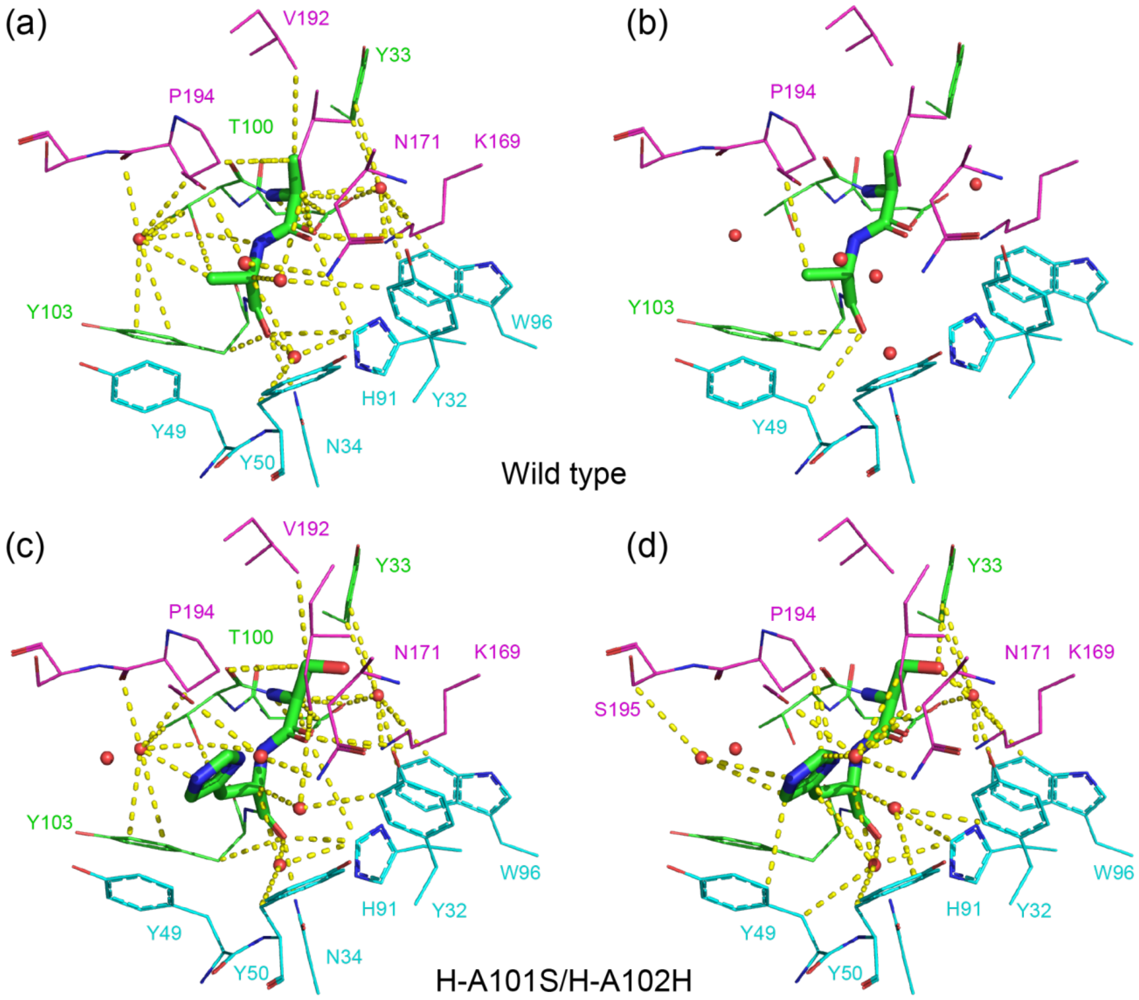

Figure S4. Interactions in the wild-type (WT; PDB ID: 1JPS[10]) and the double-point mutant model (H-A101S/H-A102H). (a) Interactions common to the WT and the mutant are depicted in the WT structure. (b) Interactions specific to the WT are depicted in the WT structure. (c) Interactions common to the WT and the mutant are depicted in the mutant structure. (d) Interactions specific to the mutant are depicted in the mutant structure. Light (L) chain, heavy (H) chain, and antigen are colored in green, cyan, and magenta, respectively. Interactions are depicted as yellow dashed lines. Water molecules are depicted as red spheres.

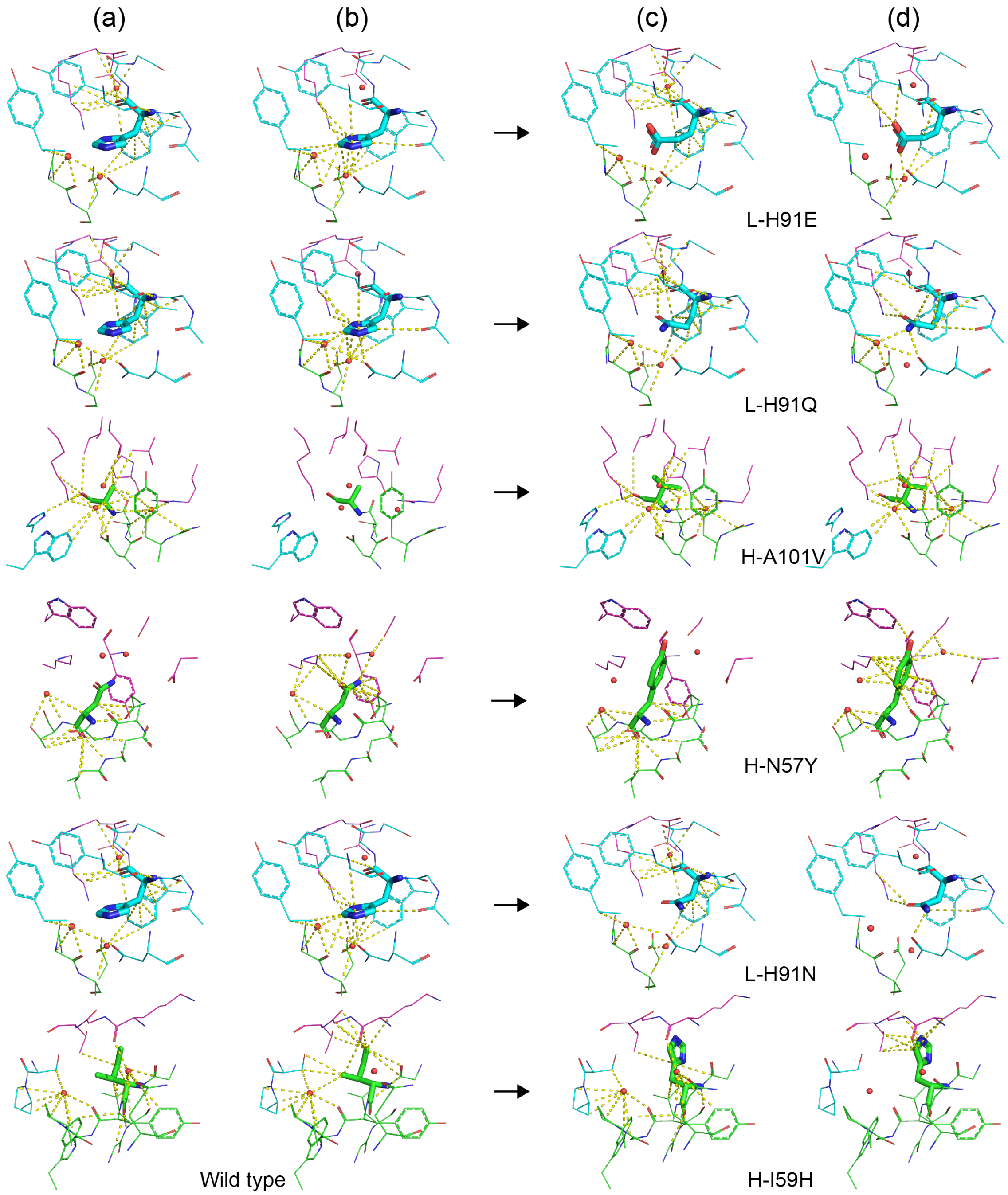

Figure S5. Interactions in the wild-type (WT; PDB ID: 1JPS[10]) and the single-point mutant models. (a) Interactions common to the WT and the mutant are depicted in the WT structure. (b) Interactions specific to the WT are depicted in the WT structure. (c) Interactions common to the WT and the mutant are depicted in the mutant structure. (d) Interactions specific to the mutant are depicted in the mutant structure. Light (L) chain, heavy (H) chain, and antigen are colored in green, cyan, and magenta, respectively. Interactions are depicted as yellow dashed lines. Water molecules are depicted as red spheres.

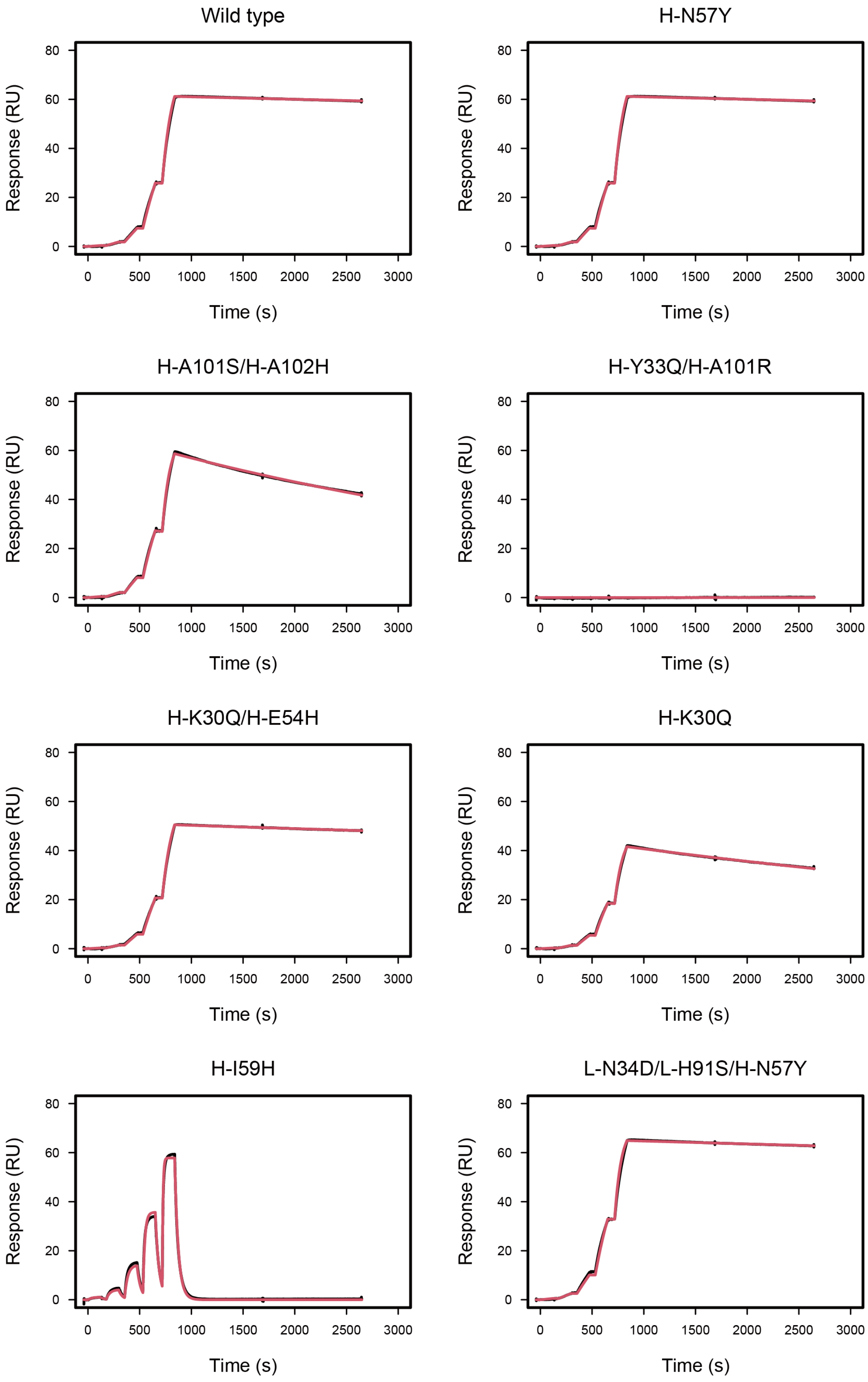
Wild type
H-N57Y
H-A101S/H-A102H
H-Y33Q/H-A101R
H-K30Q/H-E54H
H-K30Q
H-I59H
L-N34D/L-H91S/H-N57Y
Response (RU)
Time (s)
0
20
40
60
80
0
500
1000
1500
2000
2500
3000

(Continued)

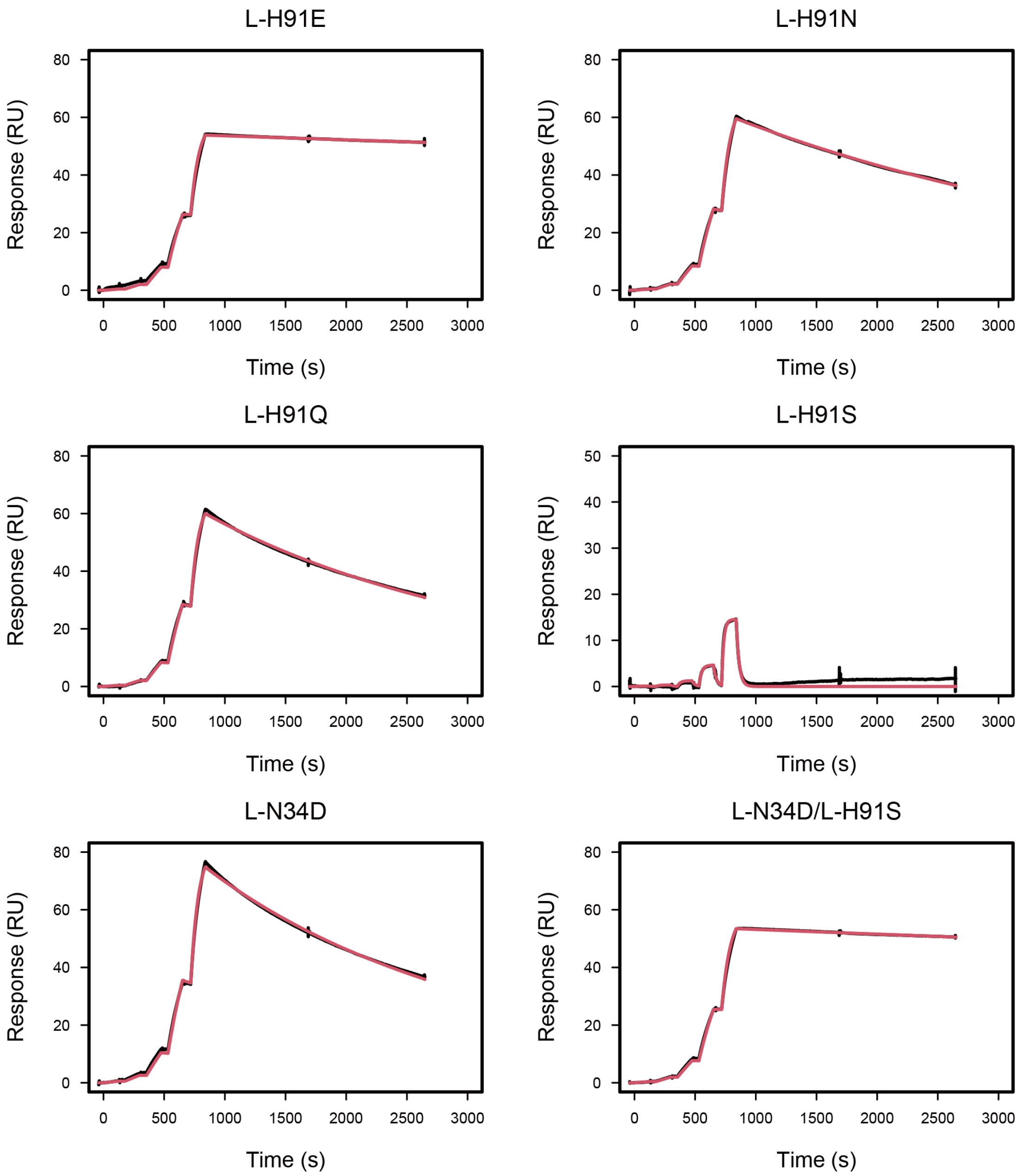

Figure S6. Single-cycle kinetics sensorgrams of the interactions of 1JPS wild-type and mutants with tissue factor (TF) by surface plasmon resonance (SPR) analysis. The concentrations of each Fab used in the analyses are 0.635 nM, 1.25 nM, 2.5 nM, 5 nM, and 10 nM. Experimental data and best fitting curves are shown in black and red, respectively.

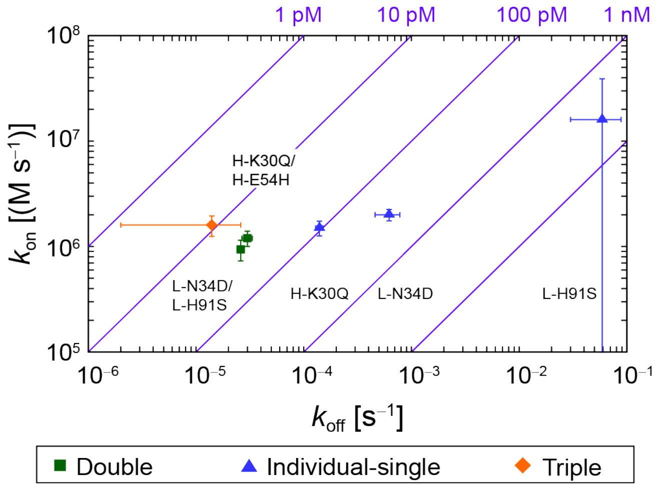

Figure S7. Binding kinetic parameters of the mutants measured by surface plasmon resonance (SPR), where the dissociation and association rate constants, k_{off} and k_{on} are plotted with error bars representing the standard deviations of three independent measurements. The purple diagonal lines show the contours of the dissociation constant, $K_D = k_{off} / k_{on}$. The measured data are presented in Table 1.

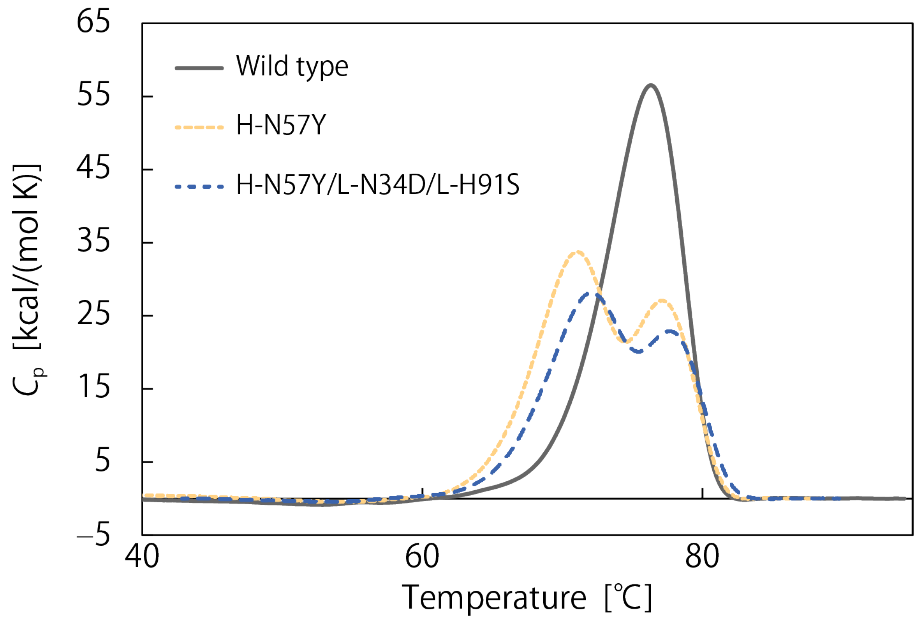

Figure S8. Heat capacity (C_p) as a function of temperature measured by differential scanning calorimetry (DSC). DSC curves of mutants studied typically showed a single melting transition peak, similar to the wild-type antibody. However, when the H-N57Y mutation was present, the DSC curve showed two peaks.

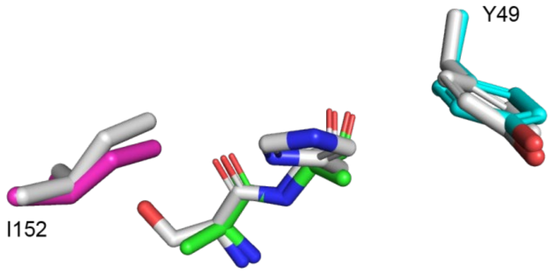

Figure S9. Conformational changes of the antigen and the light chain of the antibody after the mutation of A102 of the heavy chain of the antibody to H102. Crystallographic structure (PDB: 1JPS): heavy chain A101 and A102 (green), light chain Y49 (cyan), and I152 of the antigen (magenta). The double-point (DP) mutant model of H-A101S/H-A102H (white).

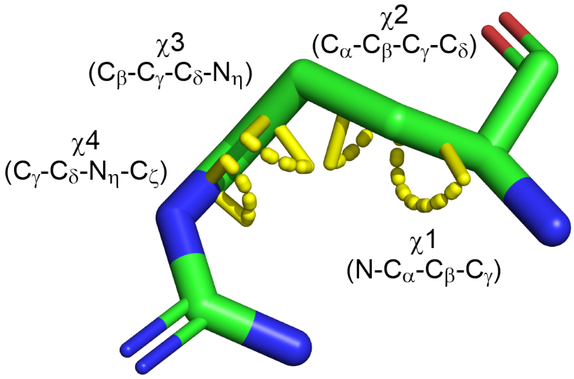

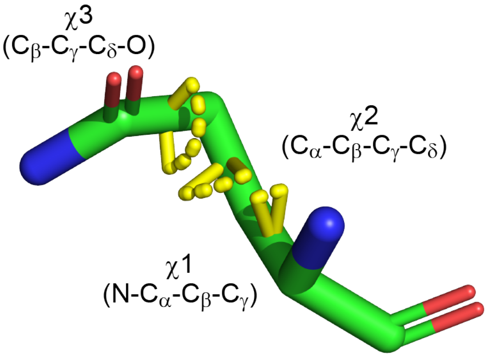

Figure S10. Conformation of the H-Y33Q/H-A101R mutant and definition of dihedrals. The conformation of the side chain of H-Q33 of the mutant was $\chi1$=gauche- (g-), $\chi2$=g- and $\chi3$=nitrogen gauche- (Ng-) and the probability of this side chain conformation in Gln was 3.4% of all 54 conformations of Gln according to the rotamer library of amino acids[11]. The maximum probability among the 54 conformations of Gln is 18.4% ($\chi1$=g-, $\chi2$=trans (t), $\chi3$=Og-). The conformation of H-R101 of the mutation model was $\chi1$=t, $\chi2$=gauche+ (g+), $\chi3$=g+, $\chi4$=g- and the probability of this conformation was 0.06 % of all of 81 side chain conformations of Arg. The maximum probability among 81 conformations of Arg is 12.2% ($\chi1$=g-, $\chi2$=t, $\chi3$=g+, $\chi4$=t).

Table S3. Dihedral angles and conformation of the side chain.

Residue	Conformation				Probability (%)	Angles			
	$\chi1$	$\chi2$	$\chi3$	$\chi4$		$\chi1$	$\chi2$	$\chi3$	$\chi4$
H-Gln33	g-	g-	Ng-	--	3.4	-23	-58	121	--
H-Arg101	t	g+	g+	g-	0.063	-160	76	70	-85

t: trans, g+: gauche +, g-: gauche -, Ng-: gauche -(nitrogen)

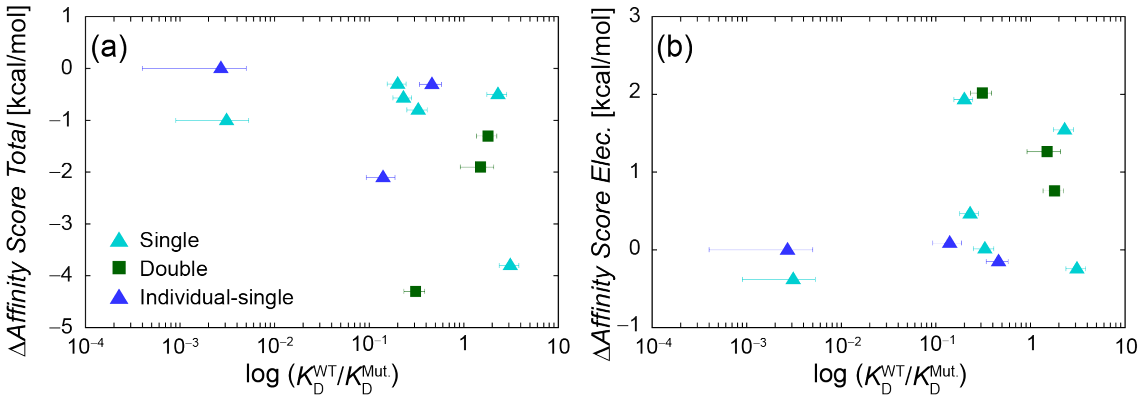

Figure S11. The plot of change of affinity score upon mutation calculated by Molecular Operating Environment (MOE) versus the experimental binding affinity change. (a) Scores calculated using a physics-based scoring function (molecular mechanics/generalized Born volume integration (MM/GBVI)) for narrowing the candidate mutants. (b) Scores calculated using electrostatic terms of the MM/GBVI scores. The affinity score changes of H-Y33Q/H-A101R, which lost binding ability completely, were -1.9 and 0.24 kcal/mol for plots a and b, respectively.

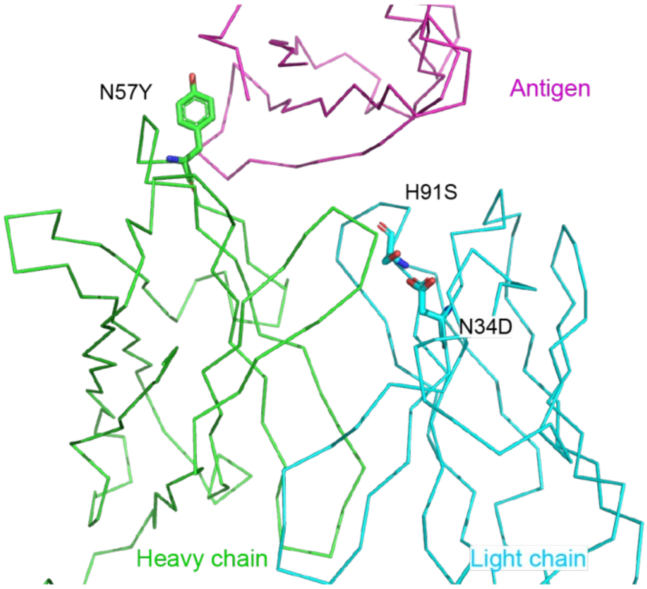

Figure S12. Triple-point mutant model (H-N57Y/L-N34D/L-H91S). The mutated residues are depicted as sticks. The distances between the N57Y and H91S and N57Y and N34D measured using Cα atoms are 17 Å and 23 Å, respectively.

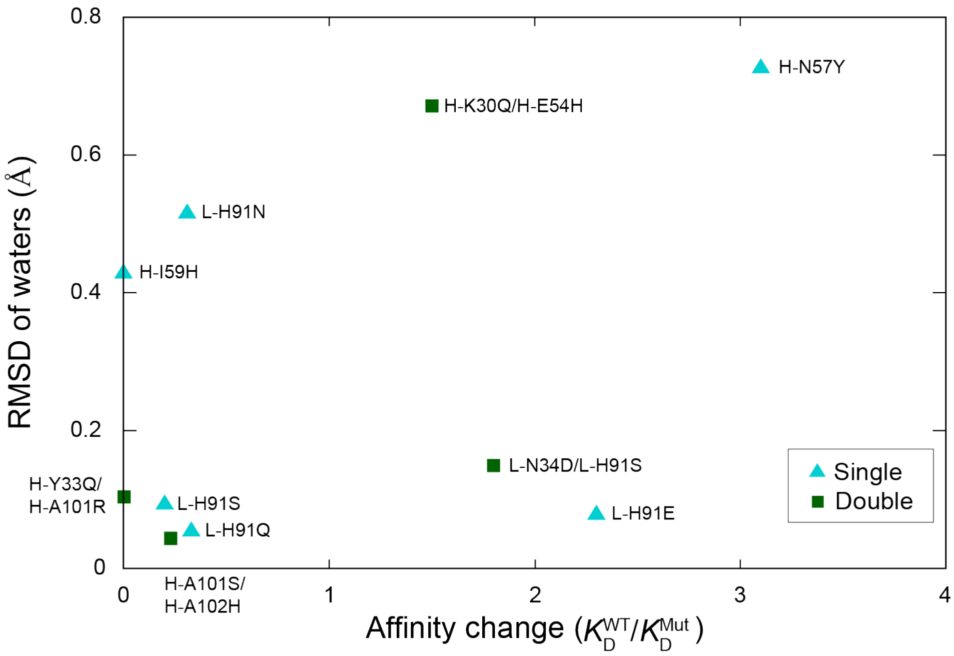

Figure S13. The root-mean-square deviations (RMSD) of oxygen of water molecules within 4.5 Å from the mutated residues between the mutant and wild-type structures. Before the RMSD calculation, the mutant and wild-type structures were aligned using atoms within 4.5 Å from the mutated residues but excluding water molecules.

(a)

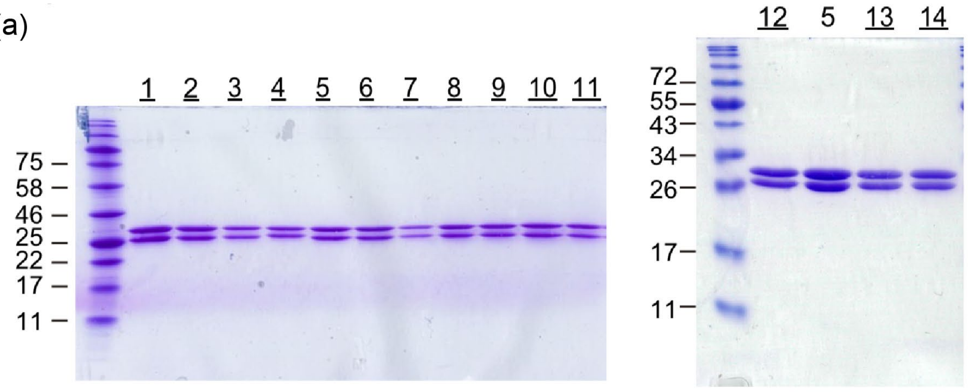

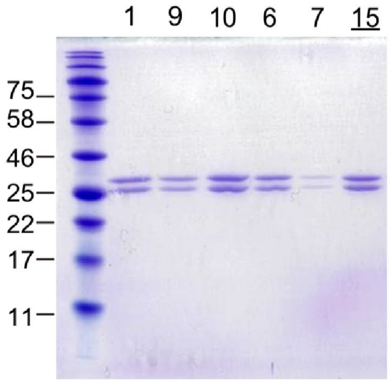

1. Wild type
2. L-H91N
3. L-H91Q
4. L-H91E
5. L-N34D/L-H91S
6. H-I59H
7. H-N57Y
8. H-K30Q/H-E54H
9. H-A101V
10. H-A101S/H-A102H
11. H-Y33Q/H-A101R
12. L-N34D/L-H91S/H-N57Y
13. L-H91S
14. L-N34D
15. H-K30Q

(b)

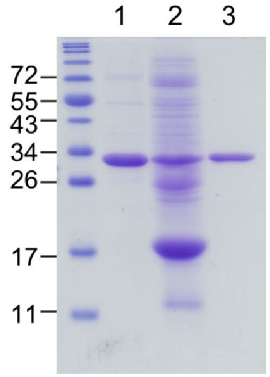

1. Flowthrough (TF sample for SEC)
2. Eliminated SUMO tag and other impurities
3. TF after final SEC purification

Figure S14. (a) The purity of obtained 1JPS wild-type (WT) and mutants was confirmed by sodium dodecyl sulfate polyacrylamide gel electrophoresis. Each gel was stained with Coomassie brilliant blue. (b) After immobilized metal affinity chromatography (IMAC) purification of tissue factor (TF), the eluted sample was dialyzed with size-exclusion chromatography (SEC) buffer and incubated with SUMO protease (Ulp1). The cleaved 6xHis-SUMO tag was collected by IMAC. Flow-through fraction (i.e. no-His tag TF) was loaded onto lane 1, and cleaved SUMO tag and other impurities are loaded onto lane 2. Lane 3 represents the TF sample obtained after further purification by SEC.

References

1 Taylor, R. & Kennard, O. Crystallographic evidence for the existence of CH...O, CH...N and CH...Cl hydrogen bonds. *J Am Chem Soc* **104**, 5063-5070, doi:10.1021/ja00383a012 (1982).

2 Nishio, M. & Hirota, M. CH/π interaction: Implications in organic chemistry. *Tetrahedron* **45**, 7201-7245 (1989).

3 Oki, M. & Iwamura, H. Steric Effects on the O-H...π Interaction in 2-Hydroxybiphenyl. *J Am Chem Soc* **89**, 576-579, doi:10.1021/ja00979a019 (1967).

4 Tsuzuki, S., Honda, K., Uchimaru, T., Mikami, M. & Tanabe, K. Origin of the Attraction and Directionality of the NH/π Interaction: Comparison with OH/π and CH/π Interactions. *J Am Chem Soc* **122**, 11450-11458, doi:10.1021/ja001901a (2000).

5 Hunter, C. A. & Sanders, J. K. M. The nature of π-π interactions. *J Am Chem Soc* **112**, 5525-5534, doi:10.1021/ja00170a016 (1990).

6 Rosenfield, R. E., Parthasarathy, R. & Dunitz, J. D. Directional preferences of nonbonded atomic contacts with divalent sulfur. 1. Electrophiles and nucleophiles. *J Am Chem Soc* **99**, 4860-4862, doi:10.1021/ja00456a072 (1977).

7 Zauhar, R. J., Colbert, C. L., Morgan, R. S. & Welsh, W. J. Evidence for a strong sulfur-aromatic interaction derived from crystallographic data. *Biopolymers* **53**, 233-248, doi:10.1002/(SICI)1097-0282(200003)53:3<233::AID-BIP3>3.0.CO;2-4 (2000).

8 Paulini, R., Muller, K. & Diederich, F. Orthogonal multipolar interactions in structural chemistry and biology. *Angew Chem Int Ed Engl* **44**, 1788-1805, doi:10.1002/anie.200462213 (2005).

9 Labute, P. The generalized Born/volume integral implicit solvent model: estimation of the free energy of hydration using London dispersion instead of atomic surface area. *J Comput Chem* **29**, 1693-1698, doi:10.1002/jcc.20933 (2008).

10 Faelber, K., Kirchhofer, D., Presta, L., Kelley, R. F. & Muller, Y. A. The 1.85 A resolution crystal structures of tissue factor in complex with humanized Fab D3h44 and of free humanized Fab D3h44: revisiting the solvation of antigen combining sites. *J. Mol. Biol.* **313**, 83-97, doi:10.1006/jmbi.2001.5036 (2001).

11 Scouras, A. D. & Daggett, V. The Dynameomics rotamer library: amino acid side chain conformations and dynamics from comprehensive molecular dynamics simulations in water. *Protein Sci* **20**, 341-352, doi:10.1002/pro.565 (2011).
